# Renal *Dnase1* expression is regulated by FGF23 but loss of *Dnase1* does not alter renal phosphate handling

**DOI:** 10.1038/s41598-021-84735-3

**Published:** 2021-03-17

**Authors:** Daniela Egli-Spichtig, Martin Y. H. Zhang, Alfred Li, Eva Maria Pastor Arroyo, Nati Hernando, Carsten A. Wagner, Wenhan Chang, Farzana Perwad

**Affiliations:** 1 Division of Nephrology, Department of Pediatrics, University of California San Francisco, San Francisco, USA; 2 Department of Medicine, San Francisco Veterans Affairs Medical Center (VAMC), University of California San Francisco, San Francisco, USA; 3 Institute of Physiology, University of Zurich, Zurich Switzerland and National Center of Competence in Research NCCR Kidney.CH, Zurich, Switzerland; 4 Children’s Renal Center, University of California San Francisco, 550, 16th Street, 5th Floor, MH-5351, San Francisco, CA 94143-3214 USA

**Keywords:** Physiology, Kidney

## Abstract

Fibroblast growth factor 23 (FGF23) is a bone-derived endocrine hormone that regulates phosphate and vitamin D metabolism. In models of FGF23 excess, renal deoxyribonuclease 1 (*Dnase1*) mRNA expression is downregulated. Dnase-1 is an endonuclease which binds monomeric actin. We investigated whether FGF23 suppresses renal Dnase-1 expression to facilitate endocytic retrieval of renal sodium dependent phosphate co-transporters (NaPi-IIa/c) from the brush border membrane by promoting actin polymerization. We showed that wild type mice on low phosphate diet and *Fgf23*^*−/−*^ mice with hyperphosphatemia have increased renal *Dnase1* mRNA expression while in *Hyp* mice with FGF23 excess and hypophosphatemia, *Dnase1* mRNA expression is decreased. Administration of FGF23 in wild type and *Fgf23*^*−/−*^ mice lowered *Dnase1* expression. Taken together, our data shows that *Dnase1* is regulated by FGF23. In 6-week-old *Dnase1*^*−/−*^ mice, plasma phosphate and renal NaPi-IIa protein were significantly lower compared to wild-type mice. However, these changes were transient, normalized by 12 weeks of age and had no impact on bone morphology. Adaptation to low and high phosphate diet were similar in *Dnase1*^*−/−*^ and *Dnase1*^+*/*+^ mice, and loss of *Dnase1* gene expression did not rescue hyperphosphatemia in *Fgf23*^*−/−*^ mice. We conclude that Dnase-1 does not mediate FGF23-induced inhibition of renal tubular phosphate reabsorption.

## Introduction

Fibroblast growth factor 23 (FGF23) is a bone-derived endocrine factor that regulates phosphate homeostasis and vitamin D metabolism^1^. In the kidney, FGF23 binds to FGF receptors 1, 3 and 4 in the presence of its obligate co-receptor, αKlotho, to inhibit renal phosphate reabsorption and suppresses 1,25 dihydroxyvitamin D (1,25(OH)_2_D) levels by inhibiting its synthesis and accelerating its degradation^2–4^. FGFR-Klotho receptor activation by FGF23 leads to phosphorylation of extracellular signaling regulated kinase (ERK 1/2). P-ERK1/2 induces the internalization of sodium phosphate co-transporter IIa (NaPi-IIa) and IIc (NaPi-IIc) from the renal brush border membrane and decreases 1,25(OH)_2_D production by downregulation of *Cyp27b1* and upregulation of *Cyp24a1* gene expression, respectively^2,5^. Further, FGF23 increases renal expression of the transcription factor, early growth response 1 (*Egr1*) via P-ERK 1/2-dependent pathway^5^. *Egr1* knockout (*Egr1*^*−/−*^) mice have similar plasma phosphate and 1,25(OH)_2_D levels as wild-type (WT) mice but the phosphaturic response to exogenous FGF23 is impaired while FGF23 mediated suppression of 1,25(OH)_2_D production is intact^6^. Hence, FGF23 regulates phosphate but not 1,25(OH)_2_D levels in an Egr-1-dependent manner^6^.

Deoxyribonuclease 1 (*Dnase1*) was among the top downregulated genes in the kidney in several studies that investigated the mechanisms by which FGF23 regulates renal phosphate and vitamin D metabolism. Specifically, comparative transcriptome analysis of Egr-1 chromatin immunoprecipitation sequencing (CHIP-seq) data of WT mice treated with FGF23 and microarray data of FGF23-treated WT mice, and *Col4a3* knockout and *Fgf23* transgenic mice revealed that FGF23 down regulates renal *Dnase1 mRNA* transcription^6,7^. FGF23 recruits Egr-1 to an active regulatory region 7.7 kb upstream of the transcription start site of the *Dnase1* gene. Intraperitoneal (i.p.) FGF23 injections suppress *Dnase1* mRNA expression in WT mice but this effect is partially blocked in *Egr1*^*−/−*^ mice^6^. Conversely, when plasma FGF23 and phosphate levels are lowered in *Hyp* and WT mice by dietary phosphate restriction, renal *Dnase1* expression is increased by several fold^8^ suggesting that *Dnase1* may be involved in FGF23-Egr-1-dependent-regulation of phosphate homeostasis.

Dnase-1 is an endonuclease which digests double stranded DNA^9^. The less recognized function of Dnase-1 is its interaction with monomeric actin (g-actin)^9^; Dnase-1 prevents actin polymerization and promotes depolymerization of filamentous actin (f-actin)^10,11^. Recently, *Dnase1* was shown to be expressed in many tissues including kidney, duodenum and liver^12^ and previous studies have shown that actin polymerization and cytoskeleton assembly is important for sodium dependent phosphate cotransporter regulation^13^. Whether FGF23 suppresses renal *Dnase1* gene expression and thereby promotes actin polymerization to facilitate endocytic retrieval of NaPi-IIa from the brush border membrane is not known. In this study, we characterized the *Dnase1*^*−/−*^ mouse model with respect to renal phosphate handling and determined whether loss of *Dnase1* gene expression rescues the hyperphosphatemic phenotype in *Fgf23*^*−/−*^ mice.

## Results

### Dietary phosphate and FGF23 regulate renal *Dnase1* gene expression

**Figure 1:**
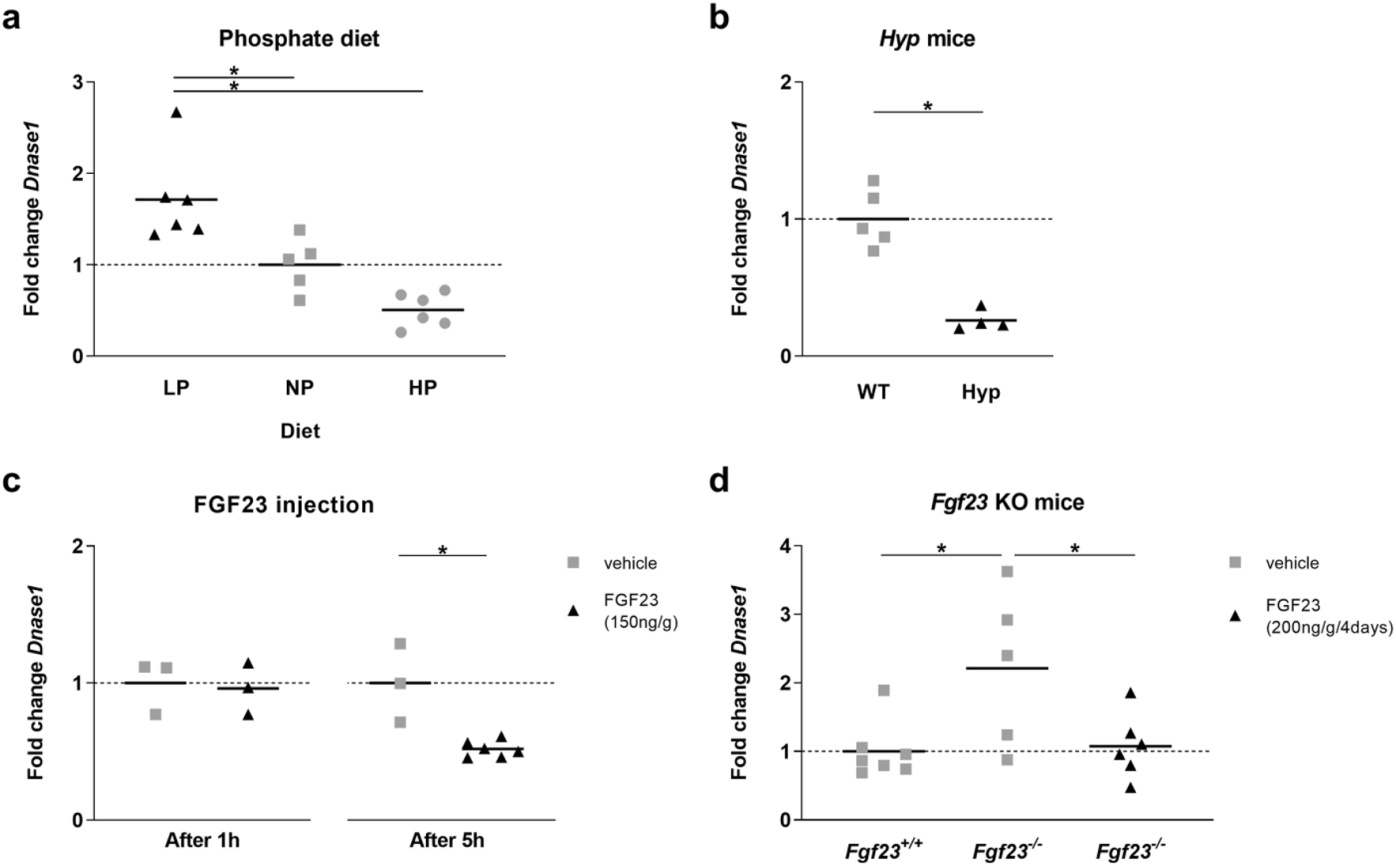
*Dnase1* mRNA expression dependent on phosphate diet and FGF23 levels. Relative renal *Dnase1* mRNA expression in (**a**) 12 week old WT mice fed for 4 days either low (black triangle), normal (gray square) or high (gray circle) phosphate diet, (**b**) in 9 weeks old WT (gray square) and *Hyp* (black triangle) mice, (**c**) in WT mice injected with a single dose of vehicle (gray square) or recombinant FGF23 (150 ng/g) (black triangle) 1 and 5 h after injection, and (**d**) in 6 week old *Fgf23*^+*/*+^ and *Fgf23*^*−/−*^ mice infused with vehicle (gray square) or recombinant FGF23 (200 ng/g) (black triangle) for 4 days. *18SrRNA* (**a**) or *Gus* (**b**–**d**) were used as housekeeping genes and values were normalized to vehicle group. 3–7 mice per group. ANOVA (**a**,**d**) with Tukey’s multiple comparisons test or Student’s t-test (**b**,**c**), *p < 0.05.

*Dnase1* mRNA expression was analyzed in WT mice fed either low (0.02%), normal (0.6%) or high (1.65%) phosphate diet for 4 days, and in mouse models of FGF23 excess and deficiency (Fig. 1a–d). In WT mice fed a low phosphate diet, *Dnase1* mRNA expression increases 1.7-fold when compared to a normal phosphate diet. In WT mice fed a high phosphate diet, *Dnase1* mRNA expression was suppressed twofold when compared to a low phosphate diet but was not significantly different from normal phosphate diet (Fig. 1a). The *Hyp* mouse is a model of FGF23 excess wherein an inactivating mutation of the *Phex* gene stimulates FGF23 production in bone thereby increasing plasma FGF23 levels inducing renal phosphate wasting and hypophosphatemia^14,15^. In *Hyp* mice, we observed that renal *Dnase1* mRNA expression was downregulated fourfold compared to WT mice (Fig. 1b). In WT mice, a single injection of recombinant FGF23 decreased renal *Dnase1* mRNA expression after 5 h (Fig. 1c). In contrast, loss of *Fgf23* gene expression in *Fgf23*^*−/−*^ mice upregulated renal *Dnase1* mRNA but this effect was reversed by infusion of recombinant FGF23 (Fig. 1d). These findings together with previously published genomic data in mice^6–8^ demonstrate the regulation of *Dnase1* mRNA expression by dietary phosphate and FGF23.

### Increased iFGF23 levels and renal phosphate leak in young ***Dnase1***^*−/−*^ mice does not impact bone health

**Figure 2:**
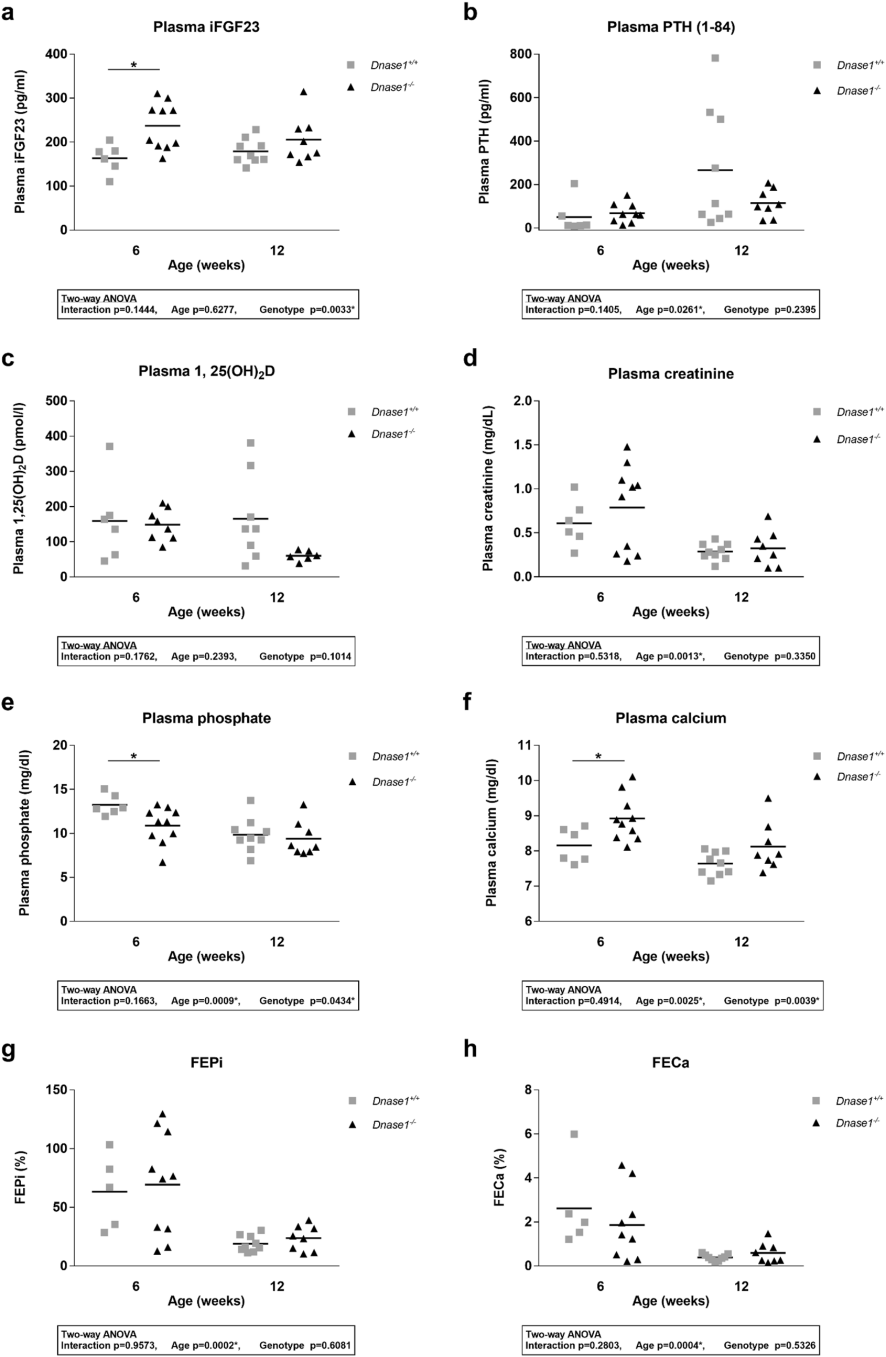
Plasma parameters of phosphate homeostasis in *Dnase1*^*−/−*^ mice. (**a**) Plasma iFGF23, (**b**) PTH (1–84), (**c**) 1,25(OH)_2_D, (**d**) creatinine, (**e**) phosphate, (**f**) calcium and fractional urinary excretion of (**g**) phosphate and (**h**) calcium in 6 and 12 week old *Dnase1*^+*/*+^ (gray square) and *Dnase1*^*−/−*^ (black triangle) mice. 5–10 mice per group. Two-way ANOVA with Sidak’s multiple comparisons test between genotypes, *p < 0.05.

**Figure 3:**
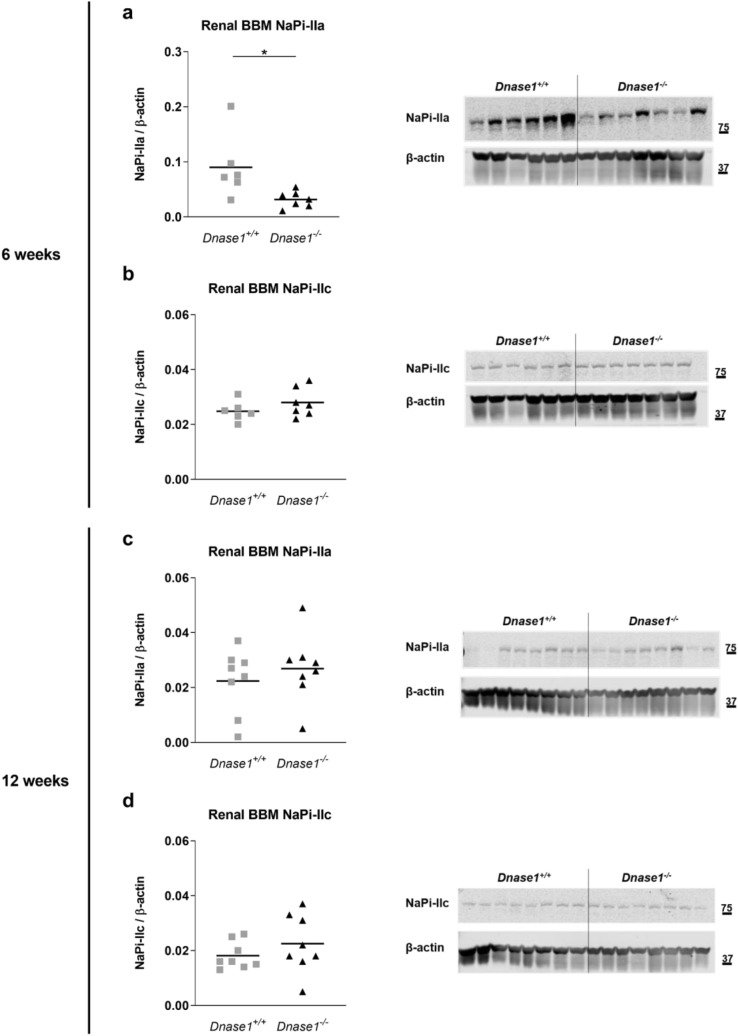
Abundance of the sodium dependent phosphate cotransporter NaPi-IIa and NaPi-IIc at the renal BBM of *Dnase1*^*-/-*^ mice. (**a**,**c**) NaPi-IIa and (**b**,**d**) NaPi-IIc protein abundance at the renal BBM relative to β-actin in 6 (**a**,**b**) and 12 (**c**,**d**) week old *Dnase1*^+*/*+^ (gray square) and *Dnase1*^*−/−*^ (black triangle) mice. 6–8 mice per group. Student’s t-test, *p < 0.05. Full-length blots are presented in the Supplementary File 2.

**Figure 4:**
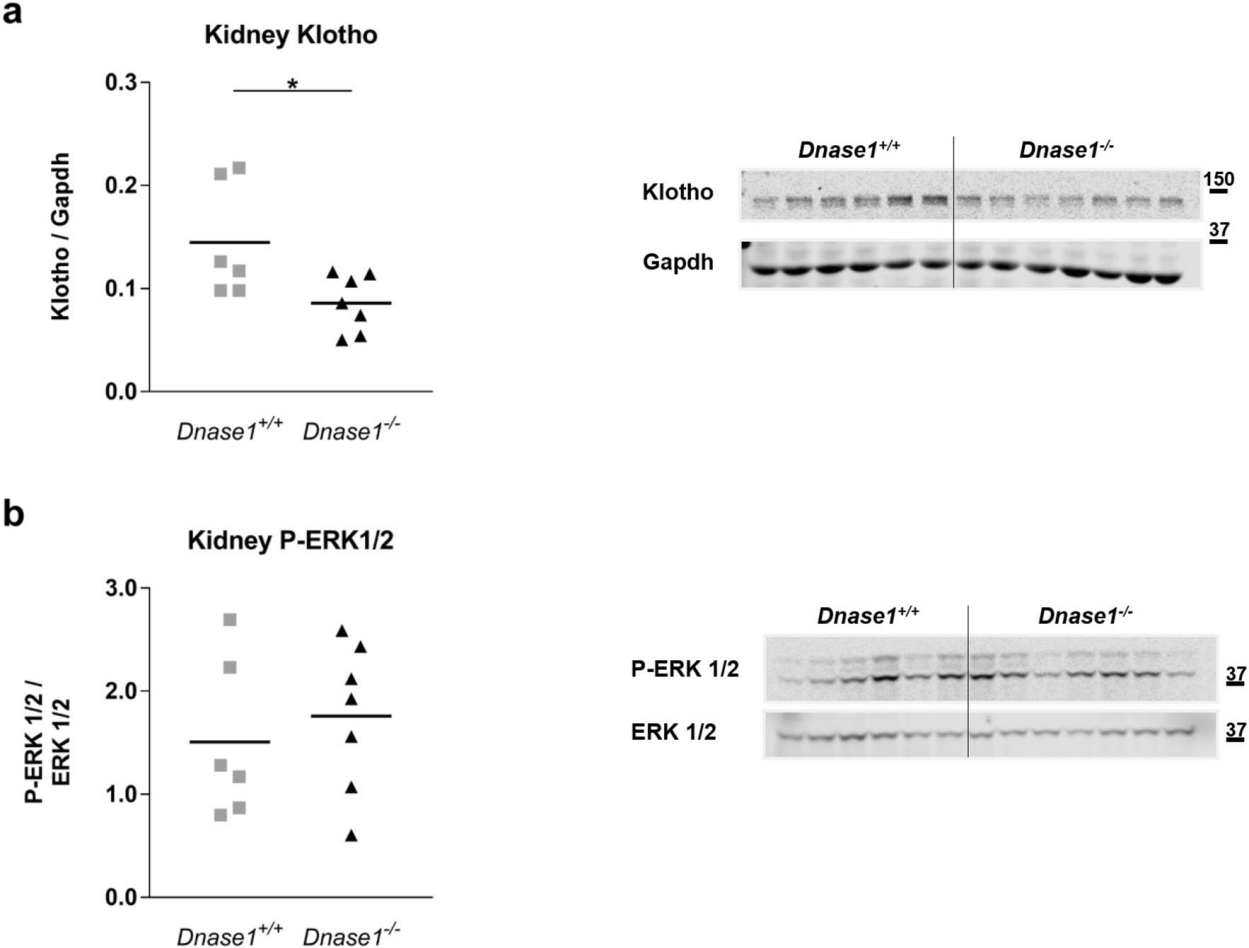
Klotho protein expression and ERK1/2 phosphorylation in kidneys of *Dnase1*^*−/−*^ mice. (**a**) Renal Klotho protein expression relative to β-actin and (**b**) ERK1/2 phosphorylation relative to total ERK1/2 in 6 week old *Dnase1*^+*/*+^ (gray square) and *Dnase1*^*−/−*^ (black triangle) mice. 6–7 mice per group. Student’s t-test, *p < 0.05. Full-length blots are presented in the Supplementary File 2.

To investigate the role of Dnase-1 in phosphate and FGF23 metabolism, we characterized the global *Dnase1*^*−/−*^ mouse model at 6 and 12 weeks of age. *Dnase1*^*−/−*^ mice were born with a normal Mendelian distribution and grew similar to their control littermates evident from normal body weight and two-kidney per bodyweight ratio (Supplementary Fig. S1). Plasma iFGF23 increased significantly by 45% in 6 week old *Dnase1*^*−/−*^ mice compared to *Dnase1*^+*/*+^ mice whereas there was no change in plasma cFGF23, PTH, 1,25(OH)_2_D and creatinine levels (Fig. 2a–d and Supplementary Fig. S2). Higher FGF23 levels in 6 week old *Dnase1*^*−/−*^ mice resulted in lower plasma phosphate but urinary fractional excretion of phosphate was unchanged. Serum calcium levels were also higher in 6 week old *Dnase1*^*−/−*^ mice but urinary fractional excretion of calcium was unchanged (Fig. 2e–h). *Fgf23*, *Galnt3* and *Fam20c* mRNA expression in bone were similar in *Dnase1*^*−/−*^ compared to *Dnase1*^+*/*+^ mice (Supplementary Fig. S2). In 12 week old *Dnase1*^*−/−*^ mice, there was no difference in plasma and urine parameters compared to *Dnase1*^+*/*+^ mice (Fig. 2). BUN levels were similar in 12 week old *Dnase1*^*−/−*^ mice compared to *Dnase1*^+*/*+^ mice suggesting normal renal function (Supplementary Fig. S3). In 6 week old *Dnase1*^*−/−*^ mice, the changes in FGF23 and phosphate levels were accompanied by decreased abundance of sodium dependent phosphate co-transporter NaPi-IIa, but not NaPi-IIc at the renal BBM compared to *Dnase1*^+*/*+^ mice (Fig. 3a,b). Localization of NaPi-IIa at the apical membrane was confirmed by immunofluorescence and was unchanged in *Dnase1*^*−/−*^ compared to *Dnase1*^+*/*+^ mice (Supplementary Fig. S4). In 12 week old *Dnase1*^*−/−*^ mice there was no change in abundance of NaPi-IIa and NaPi-IIc at the renal BBM compared to *Dnase1*^+*/*+^ mice (Fig. 3c,d). Renal Klotho protein expression was reduced in 6 week old *Dnase1*^*−/−*^ mice compared to *Dnase1*^+*/*+^ mice but ERK1/2 phosphorylation, a downstream FGF23 signaling molecule, was unchanged (Fig. 4). Furthermore, there were no changes in renal mRNA expression of *Slc34a1*, *Slc34a3*, *Cyp27b1*, *Cyp24a1*, *Klotho* and *Egr1* in 6 week old *Dnase1*^*−/−*^ compared to *Dnase1*^+*/*+^ mice (Supplementary Fig. S5). We next determined the effect of lower phosphate levels and loss of *Dnase1* expression on bone morphometry in 6 week old *Dnase1*^*−/−*^ mice. We observed that bone length, trabecular and cortical bone morphometry were not significantly different in 6 week old *Dnase1*^*−/−*^ compared to *Dnase1*^+*/*+^ mice (Table 1).

**Table 1:** Bone morphometry *Dnase1* KO mice.

	Measurement	*Dnase1*^+*/*+^	*Dnase1*^*−/−*^	t-test
		Mean ± SD	Mean ± SD	p-value
Bone length	Femur length (mm)	12.747 ± 0.214	12.560 ± 0.235	0.3052
	Tibia length (mm)	15.110 ± 0.106	15.010 ± 0.143	0.3393
Trabecular bone morphometry distal femur	BV/TV	0.110 ± 0.002	0.109 ± 0.017	0.9442
	Connectivity Density	277.424 ± 37.504	278.511 ± 27.574	0.9635
	Trabecular number	5.379 ± 0.265	5.379 ± 0.200	0.9969
	Trabecular thickness	0.034 ± 0.001	0.033 ± 0.002	0.5704
	Trabecular separation	0.186 ± 0.010	0.185 ± 0.008	0.9042
	Bone mineral density	151.502 ± 4.610	152.262 ± 20.016	0.9520
Cortical bone morphometry at tibia-fibular junction	BV/TV	0.572 ± 0.010	0.571 ± 0.016	0.9452
	Bone mineral density	758.563 ± 42.805	768.732 ± 20.061	0.6552

MicroCT Analysis of bone length, trabecular bone at distal femur and cortical bone at the tibia-fibular junction in 6-week-old *Dnase1*^+*/*+^ and *Dnase1*^*−/−*^ mice. N = 3–5 animals, SD = standard deviation, Student’s t-test.

### ***Dnase1***^*−/−*^ mice adapt appropriately to manipulation of dietary phosphate intake

**Figure 5:**
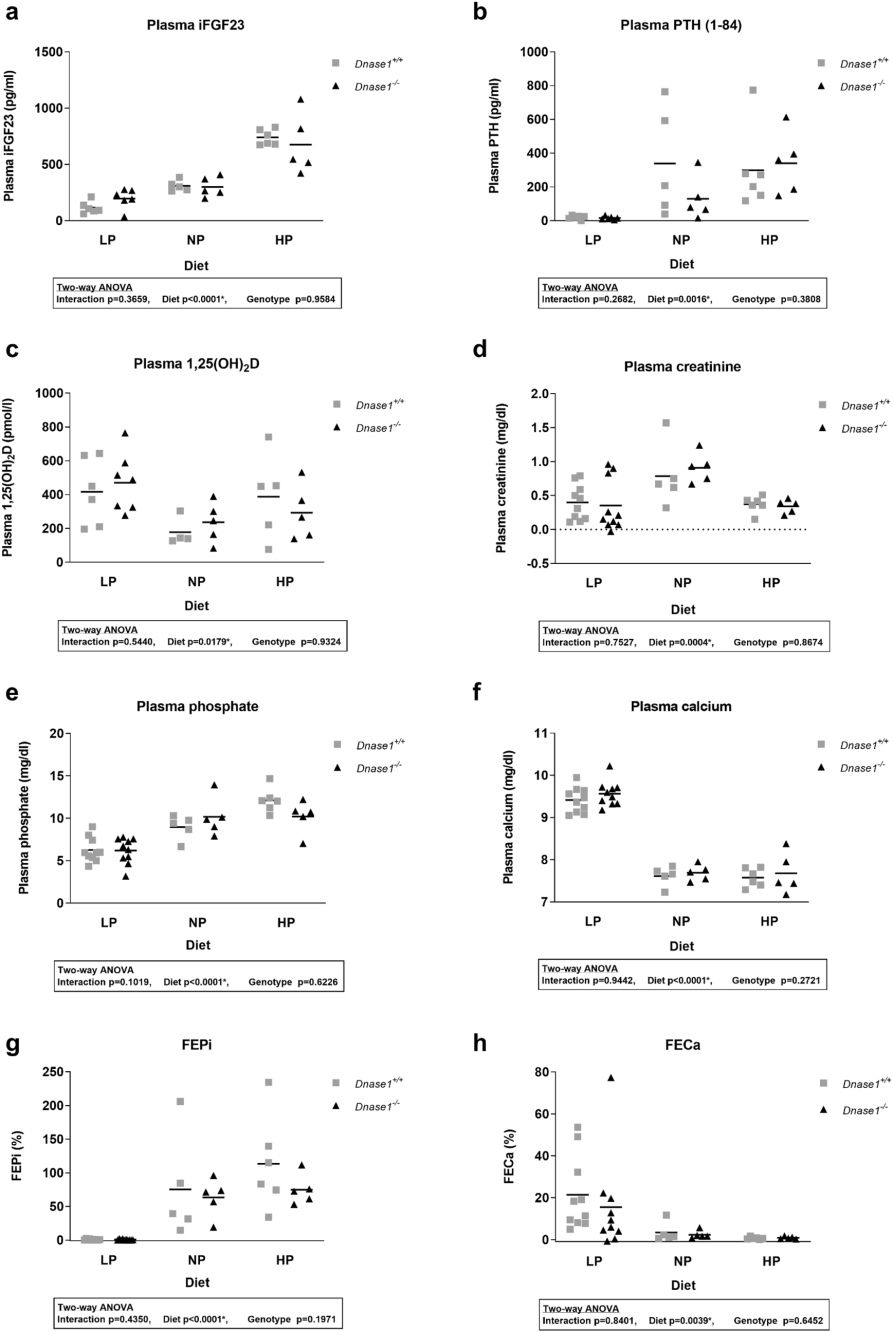
Plasma parameters of phosphate homeostasis in *Dnase1*^*−/−*^ mice dependent on phosphate diet. (**a**) Plasma iFGF23, (**b**) PTH (1–84), (**c**) 1,25(OH)_2_D, (**d**) creatinine, (**e**) phosphate, (**f**) calcium and fractional urinary excretion of (**g**) phosphate and (**h**) calcium in 12 week old *Dnase1*^+*/*+^ (gray square) and *Dnase1*^*−/−*^ (black triangle) mice fed for 4 days with low, normal or high phosphate diet. 4–10 mice per group. Two-way ANOVA with Sidak’s multiple comparisons test between genotypes, *p < 0.05.

**Figure 6:**
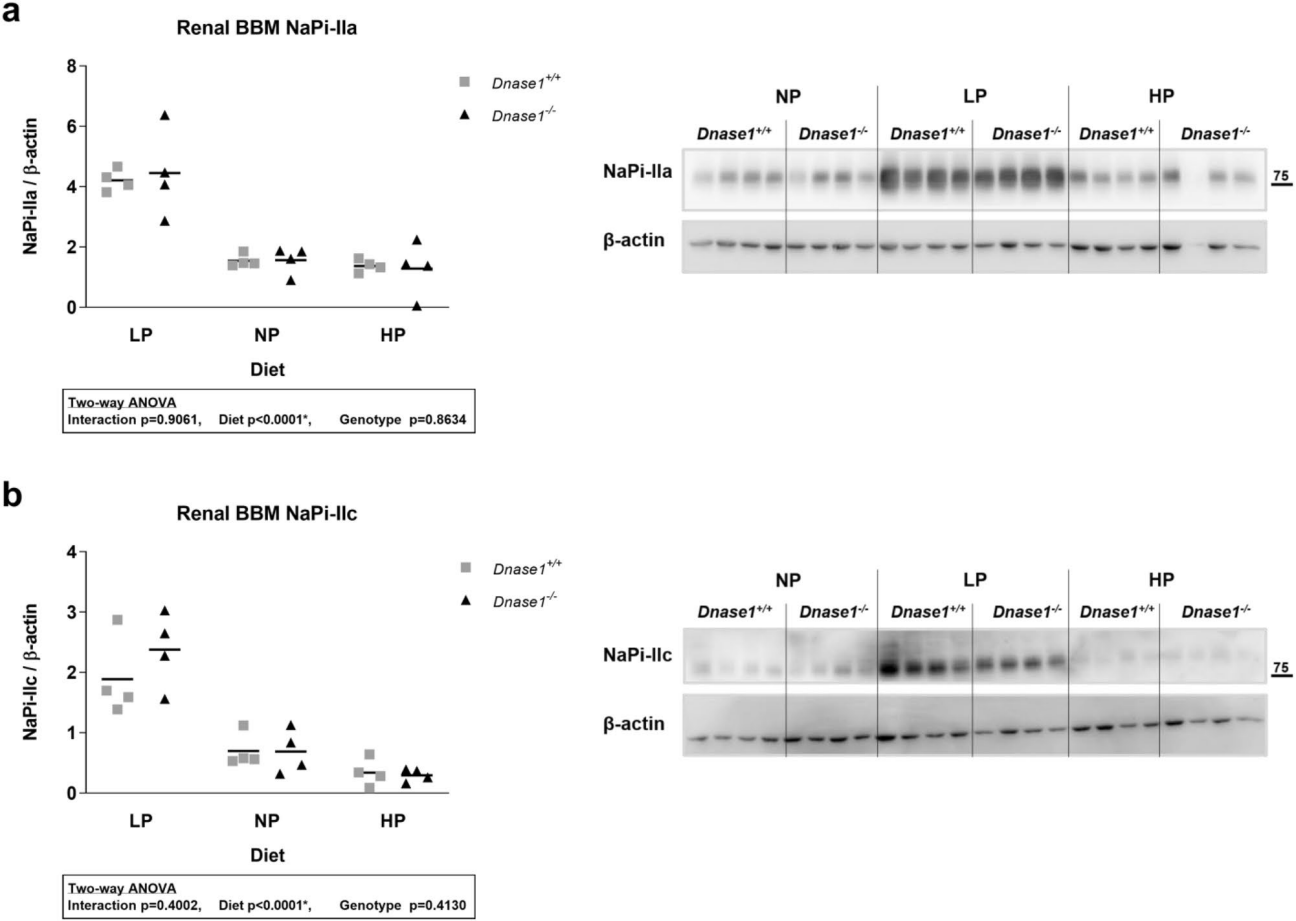
Abundance of sodium dependent phosphate co-transporter at the renal BBM of *Dnase1*^*−/−*^ mice dependent on phosphate diet. (**a**) NaPi-IIa and (**b**) NaPi-IIc protein abundance at the renal BBM relative to β-actin in 12 week old *Dnase1*^+*/*+^ (gray square) and *Dnase1*^*−/−*^ (black triangle) mice fed for 4 days with low, normal or high phosphate diet. 4 mice per group. Two-way ANOVA with Sidak’s multiple comparisons test between genotypes, *p < 0.05. Full-length blots are presented in the Supplementary File 2.

**Figure 7:**
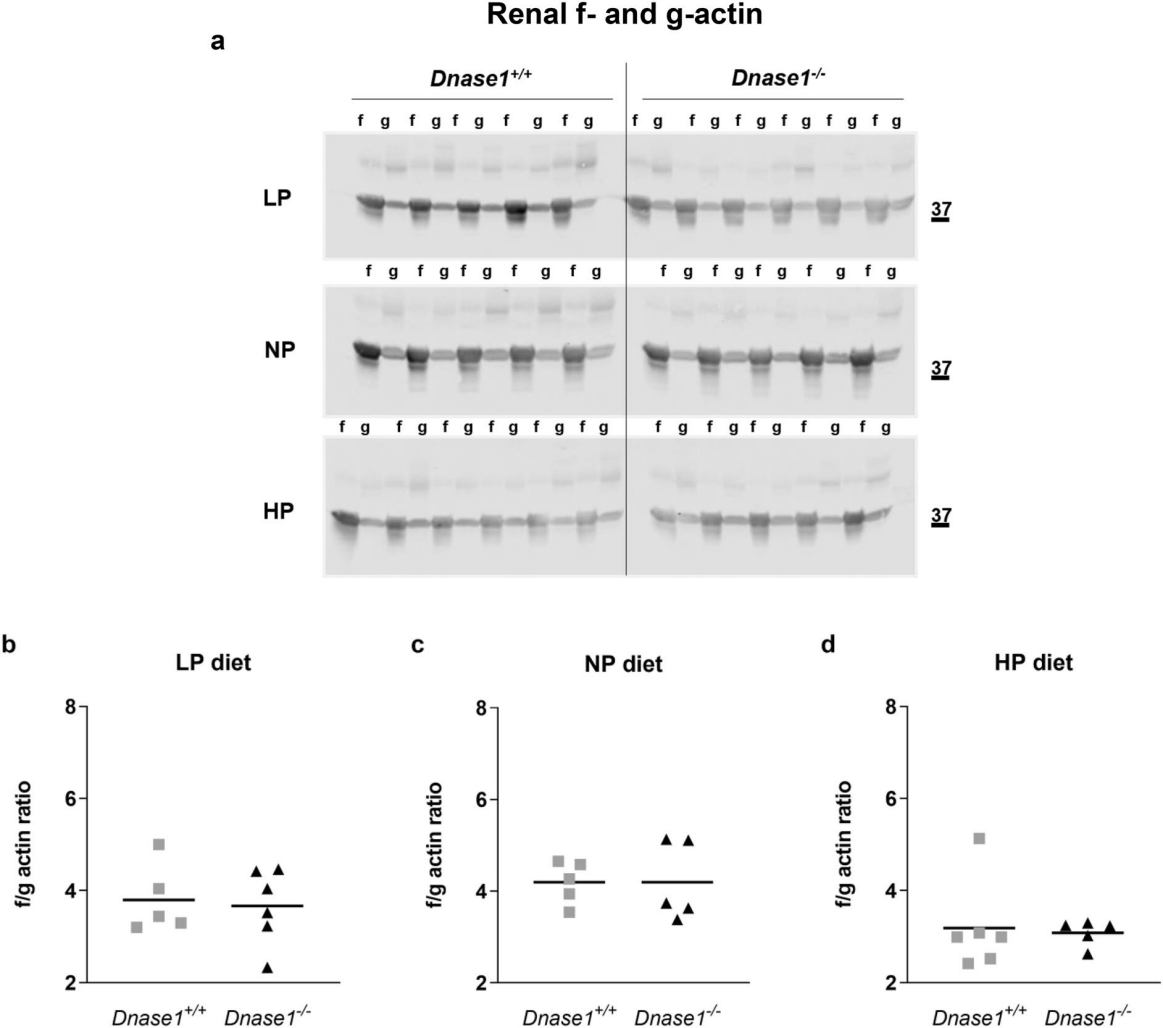
F/g actin ratio in *Dnase1*^*−/−*^ mice dependent on phosphate diet. (**a**) F- and g-actin protein abundance and (**b**–**d**) f/g actin ratio in kidneys of 12 week old *Dnase1*^+*/*+^ and *Dnase1*^*−/−*^ mice fed for 4 days with low, normal or high phosphate diet. 5–6 mice per group. Student’s t-test, *p < 0.05. Full-length blots are presented in the Supplementary File 2.

We determined whether loss of *Dnase1* gene expression has any effect on adaptation to low (0.02%) and high (1.65%) phosphate diet in 12 week old mice. We analyzed plasma iFGF23, PTH, 1,25(OH)_2_D, creatinine, phosphate, calcium as well as urinary excretion of phosphate and calcium but found no differences in adaptation to low or high phosphate diet in *Dnase1*^*−/−*^ compared to *Dnase1*^+*/*+^ mice (Fig. 5). Further there were no differences in abundance of the sodium phosphate co-transporters NaPi-IIa and NaPi-IIc at the renal BBM between *Dnase1*^*−/−*^ and *Dnase1*^+*/*+^ mice fed the same diet although *Slc34a1* mRNA expression was significantly increased by 25% in *Dnase1*^*−/−*^ compared to *Dnase1*^+*/*+^ mice fed a low phosphate diet (Fig. 6 and Supplementary Fig. S6). No changes were observed in renal mRNA expression of *Slc34a3*, *Cyp27b1*, *Cyp24a1* and *Egr1* in *Dnase1*^*−/−*^ compared to *Dnase1*^+*/*+^ mice fed the same diet (Supplementary Fig. S6). *Dnase1*^*−/−*^ mice fed a low phosphate diet had a significant increase in renal *Klotho* mRNA expression by 34% compared to *Dnase1*^+*/*+^ mice but Klotho protein abundance in the kidney between *Dnase1*^*−/−*^ and *Dnase1*^+*/*+^ mice was unchanged (Supplementary Figs. S6 and S7). Further f- to g-actin ratio was similar between *Dnase1*^*−/−*^ and *Dnase1*^+*/*+^ mice fed the same diet (Fig. 7).

### Loss of ***Dnase1*** does not correct high plasma phosphate levels in ***Fgf23***^*−/−*^ mice

**Figure 8:**
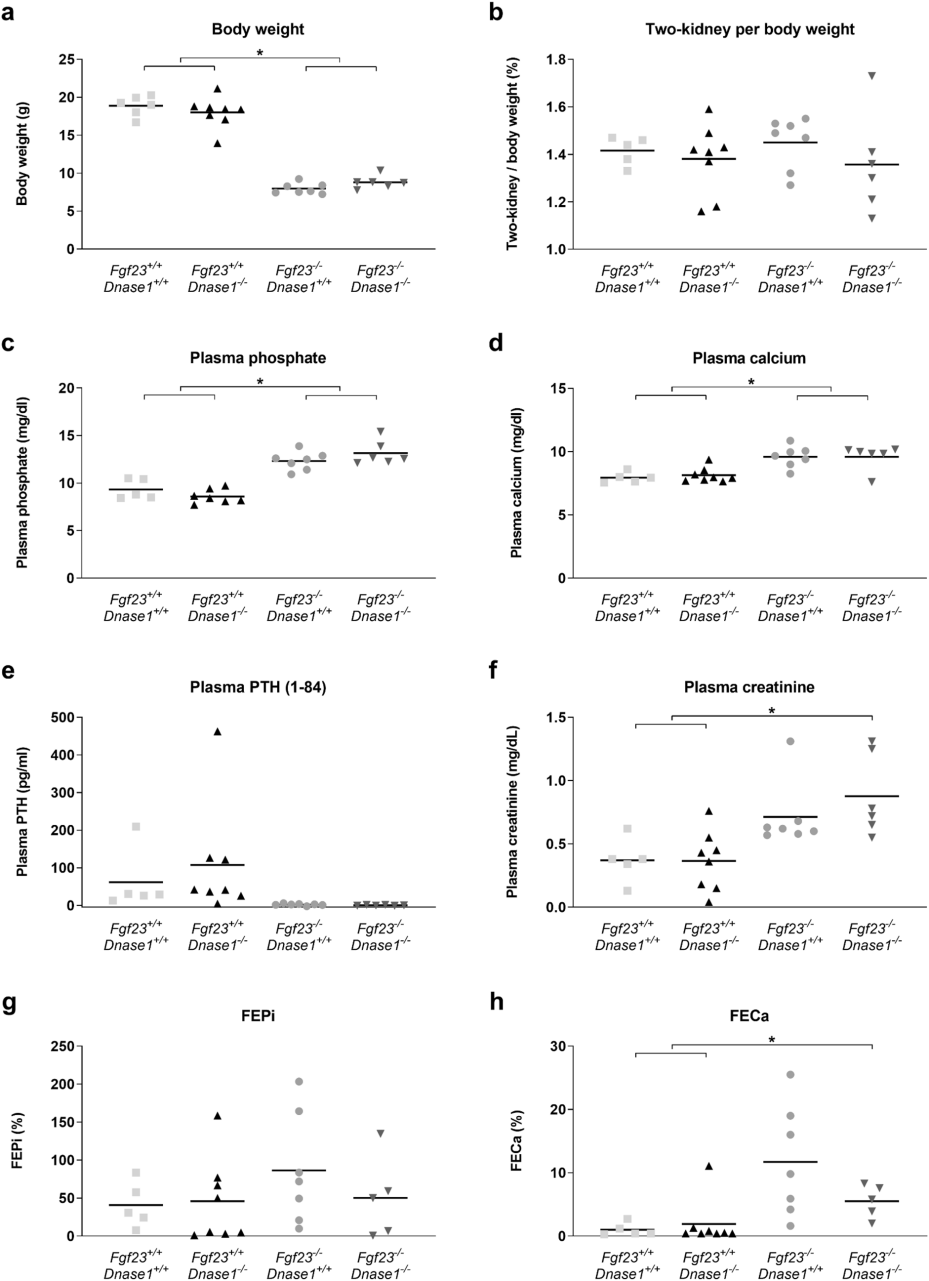
Bodyweight and plasma parameters of phosphate homeostasis in *Dnase1*^*−/−*^/*Fgf23*^*−/−*^ double KO mice. (**a**) Body weight and (**b**) two-kidney per body weight ratio, plasma (**c**) phosphate (1–84), (**d**) calcium, (**e**) PTH, (**f**) creatinine and fractional urinary excretion of (**g**) phosphate and (**h**) calcium in 6 week old *Fgf23*^+/+^, *Dnase1*^+*/*+^ (gray square), *Fgf23*^+/+^*, Dnase1*^*−/−*^ (black triangle), *Fgf23*^*−/−*^, *Dnase1*^+*/*+^ (gray circle), *Fgf23*^*−/−*^, *Dnase1*^*−/−*^ mice (black invert triangle). 5–8 mice per group. ANOVA with Tukey’s multiple comparisons test, *p < 0.05.

We evaluated the effect of loss of *Dnase1* expression in *Fgf23*^*−/−*^ mice on phosphate homeostasis in 6 weeks old *FGF23*^*−/−*^*/Dnase1*^*−/−*^ double knockout mice. *Fgf23*^*−/−*^ mice develop hyperphosphatemia as early as 10 days after birth due to increased renal tubular reabsorption of phosphate by NaPi-IIa^16^. Loss of *Dnase1* in *Fgf23*^*−/−*^ mice had no effect on body weight, two kidneys per body weight ratio, plasma phosphate, calcium, PTH and creatinine, nor on urinary fractional excretion of phosphate or calcium (Fig. 8).

## Discussion

FGF23 is a key player in phosphate homeostasis and vitamin D metabolism but little is known about intracellular mechanisms of regulation of renal tubular phosphate reabsorption by FGF23. In this study, we confirmed that FGF23 and high phosphate diet downregulate the renal expression of the actin binding protein, Dnase-1, but loss of *Dnase1* does not alter regulation of phosphate homeostasis by FGF23 in mice nor does it prevent the hyperphosphatemic phenotype of *Fgf23*^*−/−*^ mice.

Dnase-1 is a monomeric-actin binding endonuclease, which prevents polymerization of g-actin and promotes depolarization of f-actin^9–11^. We confirmed that renal *Dnase1* mRNA expression in WT mice is up- and down- regulated with low or high phosphate diet, respectively. We further demonstrate that despite the hypophosphatemia, *Dnase1* mRNA expression in *Hyp* mice is downregulated as previously described^8^ and despite the prevalent hyperphosphatemia, *Dnase1* mRNA expression is upregulated in *Fgf23*^*−/−*^ mice and is normalized by FGF23 infusion. Our findings suggest that plasma FGF23 concentration per se regulates renal *Dnase1* mRNA expression and the effect of dietary phosphate is mediated via changes in circulating FGF23. These observations confirm the results of a comparative transcriptome analysis where *Dnase1* was one of the most downregulated genes in three mouse models of FGF23 excess, the *Col4a3* knockout mouse, *FGF23* transgenic mouse and the *Hyp* mouse^7^. Together, these data clearly demonstrate that *Dnase1* mRNA expression is regulated by FGF23.

We have previously shown by ChIP-seq analysis that downregulation of *Dnase1* mRNA expression in the kidney by FGF23 is mediated by the transcription factor Egr-1^6^. Furthermore, *Egr1*^*−/−*^ mice have reduced ability to regulate plasma phosphate levels and fail to suppress renal *Dnase1* mRNA expression in response to FGF23 treatment^6^. These findings suggested that Dnase-1 plays a role in FGF23-dependent regulation of phosphate homeostasis. To analyze the potential role of *Dnase1* in the kidney to regulate tubular phosphate transport, we characterized the global *Dnase1*^*−/−*^ mouse model. Of note, deletion of *Dnase1* gene in our animal model did not interfere with tumor necrosis factor receptor-associated protein 1 (*Trap1*) gene, which shares 53 base pairs with the untranslated region of the 3′ end of *Dnase1* gene as previously reported^17,18^. *Dnase1*^*−/−*^ mice were born with a normal Mendelian distribution and did not have an obvious phenotype during the time points studied. Interestingly, we observed a transient change in phosphate homeostasis in 6 week old *Dnase1*^*−/−*^ mice with increased plasma iFGF23 but not cFGF23 and decreased plasma phosphate and abundance of NaPi-IIa at the brush border membrane. For unknown reasons, there was no change in urinary fractional excretion of phosphate despite elevated plasma iFGF23 and low plasma phosphate levels. We speculate that other phosphate cotransporters may mediate increased phosphate reabsorption to compensate for the decreased abundance of NaPi-IIa in response to hypophosphatemia. For example, it has been shown that rats at weaning age have higher levels of NaPi-IIc protein compared to adult rats^19^. NaPi-IIc significantly contributes to phosphate transport in the weaning but not adult rats as shown by injection of renal poly(A) + RNA of weaning and adult rats to oocytes and depleting NaPi-IIa or NaPi-IIc by antisense strands^19^. We did not see a change in NaPi-IIc mRNA or protein at the ages studied but it is possible there may be changes in other phosphate cotransporter activities that compensate for the reduction in NaPi-IIa. Alternatively, hypophosphatemia maybe due to reduced phosphate absorption in the intestine or increased phosphate uptake in bone, however, we did not observe any changes in bone morphometry. At 12 weeks of age, phosphate homeostasis normalized when compared to WT littermates. We challenged *Dnase1*^*−/−*^ mice with low and high phosphate diets and observed the expected changes in phosphate homeostasis depending on the diet but adaptation was similar in *Dnase1*^+*/*+^ and *Dnase1*^*−/−*^ mice. Dnase1 binds to g-actin to protect cells against untimely chromatin degradation^20^ and to modulate cytoskeleton plasticity. Cytoskeleton rearrangement is important for the regulation of sodium dependent phosphate co-transport^13^. In our study we found that loss of *Dnase1* does not impact the ratio between f- and g-actin in the kidneys of *Dnase1*^*−/−*^ mice and therefore we conclude that Dnase-1 does not affect actin polymerization or cytoskeleton rearrangement in the context of renal tubular phosphate reabsorption.

*Fgf23*^*−/−*^ mice are hyperphosphatemic with increased renal *Dnase1* mRNA expression. Loss of *Dnase1* in *Fgf23*^*−/−*^ mice did not rescue the high phosphate phenotype which suggests that the FGF23-dependent regulation of Dnase-1 is either not involved in intracellular regulation of tubular phosphate transport or an unknown compensatory mechanism overcomes the *Dnase1* deficiency in our mouse model. *Fgf23*^*−/−*^ mice demonstrate elevated calcitriol levels at an early age and^16^ calcitriol increases active transcellular phosphate uptake via NaPi-IIb in the intestine^21^. Therefore, calcitriol excess might be a dominant driver of hyperphosphatemia more than loss of FGF23 itself in *Fgf23*^*−/−*^ mice. Additionally, it may also be that the actin binding property of Dnase1 has nothing to do with regulation of phosphate homeostasis by FGF23 and the regulation of Dnase1 by FGF23 may be important for other actions of FGF23 in the kidney. Dnase1 is an endonuclease and its enzyme activity is important to remove neutrophil extracellular traps upon neutrophil activation in conditions of sterile and non-sterile inflammation^22^. Breaking up double stranded DNA by Dnase-1 prevents the formation of anti-nuclear antibodies as seen in Lupus nephritis^23^. It has been shown that Dnase1 treatment protected mice from acute lung injury (ALI) whereas *Dnase1* KO mice had worse ALI^18^. Interestingly, FGF23 has been shown to inhibit neutrophil activation, adhesion and transendothelial migration in CKD^24^. However, regulation of Dnase1 by FGF23 in the context of inflammation and reduced neutrophil activation is unknown and needs further investigation.

In conclusion, our study provides evidence for the regulation of renal Dnase-1 expression by FGF23 and transient changes in phosphate homeostasis in young *Dnase1*^*−/−*^ mice. We further demonstrate that adaptation to dietary phosphate supplementation and restriction is unaltered in *Dnase1*^*−/−*^ mice and loss of *Dnase1* expression does not rescue the high phosphate phenotype of *Fgf23*^*−/−*^ mice.

## Materials and methods

### Animals

All animal studies were approved by the Institutional animal care and use committee (IACUC), University of California San Francisco (UCSF) (approval number AN117059-02) and were in accordance with the highest ethical and animal welfare standards guidelines of IACUC, UCSF. All experiments were conducted in compliance with the ARRIVE guidelines. C57BL/6NDnase1tm1(KOMP)Vlcg/Tcp heterozygous mice (*Dnase1*^+*/−*^) were purchased from *The Center of Phenogenomics* (TCP) (Canada) and crossed with C57BL/6J mice (*Dnase1*^+*/*+^) purchased from Jackson Laboratory (USA). Heterozygous offsprings were used to generate homozygous *Dnase1*^*−/−*^ mice. Mice were housed in a controlled environment with a 12:12-h light–dark cycle and fed ad libitum with regular chow (PicoLab Mouse Diet 20 5058). Six or twelve-week-old male and female mice were used for all experiments. 24-h urine was collected in metabolic cages (Tecniplast, Italy) from 6 and 12 week old mice before they were anesthetized with Ketamine/Xylazine for subsequent blood and organ collection. For the diet study, 12 week old mice were fed for 4 days either with normal (0.6% phosphorus, 0.6% calcium, TD.09261), low (0.02% phosphorus, 0.6% calcium, TD.09262) or high (1.65% phosphorus, 0.6% calcium, TD.110217) phosphate diet (Envigo Tekland diets, USA), respectively. On the last day of the diet, spot urine samples were collected and mice were anesthetized with Ketamine/Xylazine before blood collection and tissue harvest.

Heterozygous *Fgf23*^*tm1Blan*^ (*Fgf23*^+*/−*^) mice^25^ were crossed with *Dnase1*^+*/−*^ mice to generate *Fgf23*^*−/−*^, *Dnase1*^*−/−*^ double knockout mice. Six week old *Fgf23*^*−/−*^, *Dnase1*^*−/−*^ double knockout mice, 9 week old *Hyp* mice (*B6.Cg-Phex*^*Hyp/J*^) and 6 week old *Fgf23*^*−/−*^ mice implanted with Alzet osmotic infusion pumps were used for the experiments when appropriate. The pump infused saline or 200 ng per gram body weight per day recombinant FGF23 (Genzyme-Sanofi) for 4 consecutive days. Mice were housed in a controlled environment with a 12:12-h light–dark cycle and fed ad libitum with regular chow (PicoLab Mouse Diet 5053).

### Biochemistries

Blood was collected in BD Microtainer Tubes containing Lithium Heparin (Becton, Dickinson and Company) for plasma separation. Plasma and urine were aliquoted, rapidly frozen and stored at − 80 °C. Plasma and urine phosphate, calcium and creatinine were measured with Phosphorus Liqui-UV test, Calcium (CPC) LiquiColor or Creatinine LiquiColor test, respectively (EKF Stanbio, USA). BUN was measured by Idexx BioAnalytics (West Sacramento, CA, USA). The plasma concentration of iFGF23 (Immutopics International, USA), cFGF23 (Immutopics International, USA), PTH (1–84) (Immutopics International, USA) and 1,25(OH)_2_D (Immunodiagnostic Systems Inc, UK) were measured by enzyme-linked immunosorbent assays according to manufacturers’ protocols.

### RNA extraction, reverse transcription and qPCR

Organs were harvested and rapidly frozen in liquid nitrogen. Kidneys were homogenized using a BeadBug microtube homogenizer. Total RNA from kidney was extracted with NucleoSpin RNA lysis buffer followed by purification with NucleoSpin RNA Miniprep (Clontech, USA) according to the manufacturers’ protocol including Dnase-1 digestion. Total RNA extractions were analyzed for purity and concentration using the NanoDrop ND-1000 spectrophotometer. RNA samples were diluted to a final concentration of 100 ng/μl and cDNA was prepared with reagents from Invitrogen (USA) if not stated otherwise. In brief, in a reaction volume of 40 μl, 300 ng of RNA was used as template and mixed with the following final concentrations of RT buffer (1×): MgCl_2_ (5.5 mmol/l), random hexamers (2.5 μmol/l), dNTP mix (500 μmol/l each) (Bioline Ltd., USA), RNase inhibitor (0.4 U/μl), multiscribe reverse transcriptase (1.25 U/μl), and RNAse-free water. Reverse transcription was performed with temperature conditions set at 25 °C for 10 min, 48 °C for 30 min, and 95 °C for 5 min on a thermocycler (Eppendorf). Quantitative PCR (qPCR) was performed using the ABI PRISM 7900HT Detection System (Applied Biosystems). Primers were designed using Primer 3 software^26,27^. Primers and probes were purchased either from Elim Biopharma (USA), IDT (USA) or Applied Biosystems (Eukaryotic 18S rRNA Endogenous Control primer probe set) (Table 2). Probes were labeled with the reporter dye FAM at the 5′-end and the quencher dye TAMRA or BHQ1 at the 3′-end. qPCR reactions were performed using the TaqMan Fast Advanced Master Mix or PowerUp SYBR Green Master Mix (Applied Biosystems, USA).

**Table 2:** Probe and primer sequences used for qPCR.

Gene	Sequence	
Mouse *Slc34a1*	Fwd	5′-GTCTCATTCGGATTTGGTGTCA-3′
	Rev	5′-GCCGATGGCCTCTACCCT-3′
	Probe	5′-CCAGACACAACAGAGGCTTCCACTTCTATGTC-3′
Mouse *Slc34a3*	Fwd	5′-TAATCTTCGCAGTTCAGGTTGCT-3′
	Rev	5′-CAGTGGAATTGGCAGTCTCAAG-3′
	Probe	5′-CCACTTCTTCTTCAACCTGGCTGGCATACT-3′
Mouse *Cyp27b1*	Fwd	5′-CCTCTGCCGAGACTGGGA-3′
	Rev	5′-TCCCGAAAAAGGAAGTGGGT-3′
	Probe	5′-TGTTTGCCTTTGCCCAGAGGCAC-3′
Mouse *Cyp24a1*	Fwd	5′-TACGCTGCTGTCACGGAGC-3′
	Rev	5′-TCTGGATTTCCCGGAGAAGTC-3′
	Probe	5′-CAGTGGAGACGACCGCAAACAGCTT-3′
Mouse *Klotho*	Fwd	5′-CAGCTCCAGGCTCGGGTA-3′
	Rev	5′-AGGTGTTGTAGAGATGCCAGACTTT-3′
	Probe	5′-TTGCCCACAACCTACTTTTGGCTCATG-3′
Mouse *Gus*	Fwd	5′-CTCATCTGGAATTTCGCCGA
	Rev	5′-GGCGAGTGAAGATCCCCTTC-3′
	Probe	5′-CGAACCAGTCACCGCTGAGAGTAATCG-3′
Mouse *Fgf23*	Fwd	5′-GACCAGCTATCACCTACAGATCCA-3′
	Rev	5′-CGGCGTCCTCTGATGTAATCA-3′
	Probe	5′-CCCATCAGACCATCTACAGTGCCCTGA-3′
Mouse *Egr1*	Fwd	5′-CCTATGAGCACCTGACCACA-3′
	Rev	5′-TCGTTTGGCTGGGATAACTC-3′
	Probe	Roche Universal Probe #22
Mouse *Dnase1*	Fwd	5′-ACTTTGTGAAAATCCTGAGTCGC-3′
	Rev	5′-AGCGGTAGGTGTCAGGTTTG-3′
	Probe	5′-TCCCACCTGGTTGCTGTTGGGAAGC-3′
Mouse *Galnt3*	Fwd	5′-GAGAAAGAGCGAGGGGAAAC-3′
	Rev	5′-GTGGACCATGCTTCATTGTG-3′
	Probe	5′-ACACCCGACCACCTGAATGTATTGAAC-3′
Mouse *Fam20c*	Fwd	5′-GGGAACATGGATCGGCATCA-3′
	Rev	5′-GCACTGATGAAGAGGAGCGA-3′
	Probe	5′-AACGGGCGCGGGTTTGGGAAATACT-3′

### Protein extraction and western blot

Protein extraction and western blot was performed as previously described^28,29^. Organs were rapidly frozen in liquid nitrogen. Tissues were homogenized in a BeadBug microtube homogenizer with buffer containing either 300 mM Mannitol, 5 mM EGTA, 12 mM Tris base (Sigma-Aldrich, USA) and 1 × complete protease inhibitor cocktail (Roche, USA) (pH 7.1) for brush border membrane (BBM) extraction or 20 mM Tris–HCl (pH 7.5), 150 mM NaCl, 1% NP-40, 0.5% sodium deoxycholate, 1 M EDTA, 0.1% SDS (Sigma-Aldrich, USA) and 1 × protease inhibitor cocktail (Roche, USA) for total protein extraction. Brush border membrane vesicles were prepared using the Mg^2+^-precipitation technique^30^. Protein concentration was determined with the Pierce BCA Protein Assay Kit or the DC Protein Assay from Biorad. Twenty µg of renal brush border membrane for NaPi-IIa and NaPi-IIc and 50 µg (Klotho/Dnase-1) or 35 µg (P-Erk/Erk) of total kidney protein, respectively, were solubilized in loading buffer containing DTT and separated on 8% or 10% polyacrylamide gels.

For immunoblotting, proteins were transferred electrophoretically to polyvinylidene fluoride membranes (Immobilon-P, Millipore, USA). Membranes were blocked with Odyssey Blocking Buffer (PBS) (Li-cor, USA) or 5% milk powder in Tris-buffered saline with 0.1% Tween-20 for 60 min followed by primary antibody incubation either for 2 h at room temperature or overnight at 4 °C. Primary antibodies used: rabbit polyclonal anti-NaPi-IIa (1:3000)^31,32^, rabbit polyclonal anti-NaPi-IIc (diet study) (1:750)^33^, chicken polyclonal NaPi-IIc (6 and 12 week old *Dnase1*^*−/−*^ and *Dnase1*^+*/*+^ mice) (kind gift from Dr. Moshe Levi from the University of Colorado, Denver), mouse monoclonal anti-β-actin antibody (Sigma, St. Louis, MO; 1:5000), mouse monoclonal anti-Erk1/2 (#4370, Cell Signaling Technology, USA; 1:2000), rabbit monoclonal anti-Phospho-Erk1/2 (#9107, Cell Signaling Technology, USA; 1:2000), rat monoclonal anti-Klotho (Clone KM2076, TransGenic Inc., Japan; 1:1000) and mouse monoclonal anti-GAPDH (Merck Millipore, USA; 1:2000). Membranes were incubated with secondary antibodies for 1 h at room temperature: anti-Rabbit IgG, HRP conjugate (W4018, Promega, USA), anti-Mouse IgG, HRP conjugate (W4028, Promega, USA), IRDye 800CW Goat anti-Rabbit IgG (P/N 925-32211), IRDye 680RD (P/N 925-68072), IRDye 800CW Donkey anti-Chicken IgG (P/N 925-32218), IRDye 680RD Goat anti-Mouse IgG (P/N 925-68070), IRDye 800CW Goat anti-Rat (P/N 925-32219), IRDye 680RD Goat anti-Rabbit IgG (P/N 925-68071) (all Li-cor, USA). Immobilon Western (Cat.No WBKLS0500, Millipore) was used as HRP substrate. DIANA III-chemiluminescence detection system (Raytest, Straubenhardt, Germany) or Li-cor Odyssey Classic fluorescent scanner was used for signal detection. Image contrast and brightness were adjusted equally across the entire image using Image Studio Lite from Li-cor (https://www.licor.com/bio/image-studio-lite/download?gclid=Cj0KCQiA0MD_BRCTARIsADXoopaIkX18Z5l3DwJOvtTmSeLiCm8o5xmlUjfL-z0ZOVT2h-2oVOlwNTEaAub2EALw_wcB). Signal quantification was performed solely within the same blot using the Image Studio Lite software from Li-cor.

### F- and g-actin extraction

F- and g-actin extraction was done with the G-actin/F-actin In Vivo Assay Kit (# BK037, Cytoskeleton, USA) according to the manufactures’ protocol.

### Microcomputed tomography (μCT)

Microarchitecture of the trabecular bone of the distal femur (DF), and of the cortical bone at the tibiofibular joint (TFJ), was measured using a Scanco Medical mCT 50 specimen scanner. Samples were fixed in 10% phosphate-buffered formalin and placed in 70% ethanol. Bones were scanned at an X-ray energy level of 55 kVp and intensity of 109 μA. The µCT 50 is calibrated monthly using a calibration phantom for density measurements. Trabecular bone volume fraction and microarchitecture were analyzed in the secondary spongiosa at the distal femur. The trabecular region of interest started adjacent and proximal to the femoral growth plate and extended 1 mm towards the proximal end of the femur. Cortical bone was analyzed in the mid-diaphysis of the tibia. The cortical region of interest started adjacent to the TFJ and extended proximally for 0.4 mm. Measurements included bone volume/total tissue volume (BV/TV), trabecular number (Tb.N.), trabecular thickness (Tb.Th.), and trabecular spacing (Tb.Sp). All scans were analyzed using Scanco Medical μCT evaluation software version 6.0. The evaluation script for trabecular analysis was set at Gaussian sigma of 0.5 and support of 2, and a lower threshold of 300. The script for cortical analysis was set at sigma of 0.8, support of 1, and lower threshold of 380. Representative 3D images created using Scanco Medical mCT Ray v4.0 software.

### Immunofluorescence staining

Immunofluorescence staining was performed as previously described^29^. Briefly, mouse kidneys were perfused through the left heart ventricle with a fixative solution containing 3.2% paraformaldehyde (PFA) in phosphate-buffered saline (PBS). Mouse kidneys were removed and immersed in 3.2% PFA for additional 3 h. Next, kidneys were washed with PBS and incubated overnight in 30% sucrose in PBS. Kidneys were embedded in O.C.T. media and frozen in liquid propane cooled with liquid nitrogen. Then, 5 μm cryosections were cut. Slides were rehydrated/washed in PBS before they were treated for 5 min with 0.5% sodium dodecyl sulfate in PBS. Unspecific sites were blocked with 1% bovine serum albumin in PBS for 1 h at room temperature. Primary rabbit anti-NaPi-IIa antibody (kind gift from Dr. Moshe Levi from the University of Colorado, Denver) was diluted 1:500 in 1% bovine serum albumin in PBS and kidney sections were incubated with the primary antibody overnight at 4 °C. After washing with PBS, sections were incubated with the corresponding secondary antibody (1:1000) [anti-rabbit Alexa594 (Invitrogen)], phalloidin-488 (1:250, Invitrogen), and Hoechst (1:1000, Sigma) for 1 h at room temperature. Slides were washed twice with PBS before being mounted with Dako glycergel mounting medium. Sections were visualized on a Leica DM 5000B fluorescence microscope and images processed with Leica Las X software (https://www.leica-microsystems.com/products/microscope-software/p/leica-las-x-ls/).

### Statistical analysis

Statistics were performed using unpaired Student’s t-test, ANOVA with Tukey’s multiple comparisons analysis or Two-Way-ANOVA with Sidak’s multiple comparison analysis between genotypes (GraphPad Prism version 8, GraphPad, San Diego, CA). P < 0.05 was considered significant.

## Supplementary Information

Supplementary Information 1.

Supplementary Information 2.
