# Supplementary material for: Renal Dnase1 expression is regulated by FGF23 but loss of Dnase1 does not alter renal phosphate handling
Source: Sci Rep. 2021 Mar 17;11:6175. doi: 10.1038/s41598-021-84735-3 (PMC7969776; doi:10.1038/s41598-021-84735-3)
Supplement: Supplementary file 1 — Supplementary Information 1. [file 41598_2021_84735_MOESM1_ESM.pdf]

Supplementary Figure S1

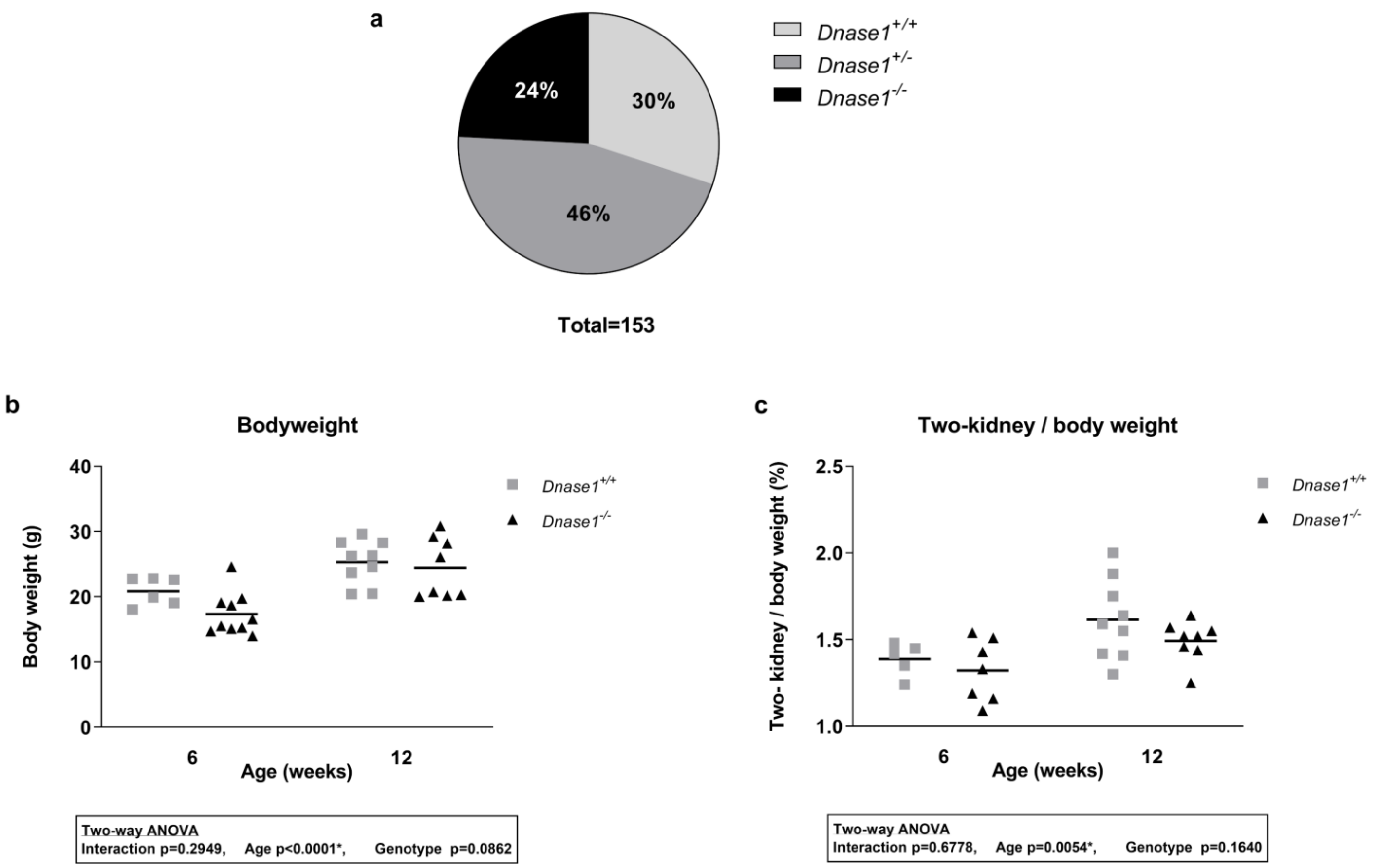

Supplementary Fig. S1. Genotype distribution, body and kidney weight of *Dnase1*$^{-/-}$ mice. Genotype of 153 pups which originate from 6 heterozygous Dnase1 breeding pairs with 3-5 litters each. (b) Body weight and (c) two-kidney per body weight ratio of 6 and 12 week old *Dnase1*$^{+/+}$ (■) and *Dnase1*$^{-/-}$ (▲) mice. 5-10 mice per group. Two-way ANOVA with Sidak's multiple comparisons test between genotypes, * $p < 0.05$.

Supplementary Figure S2

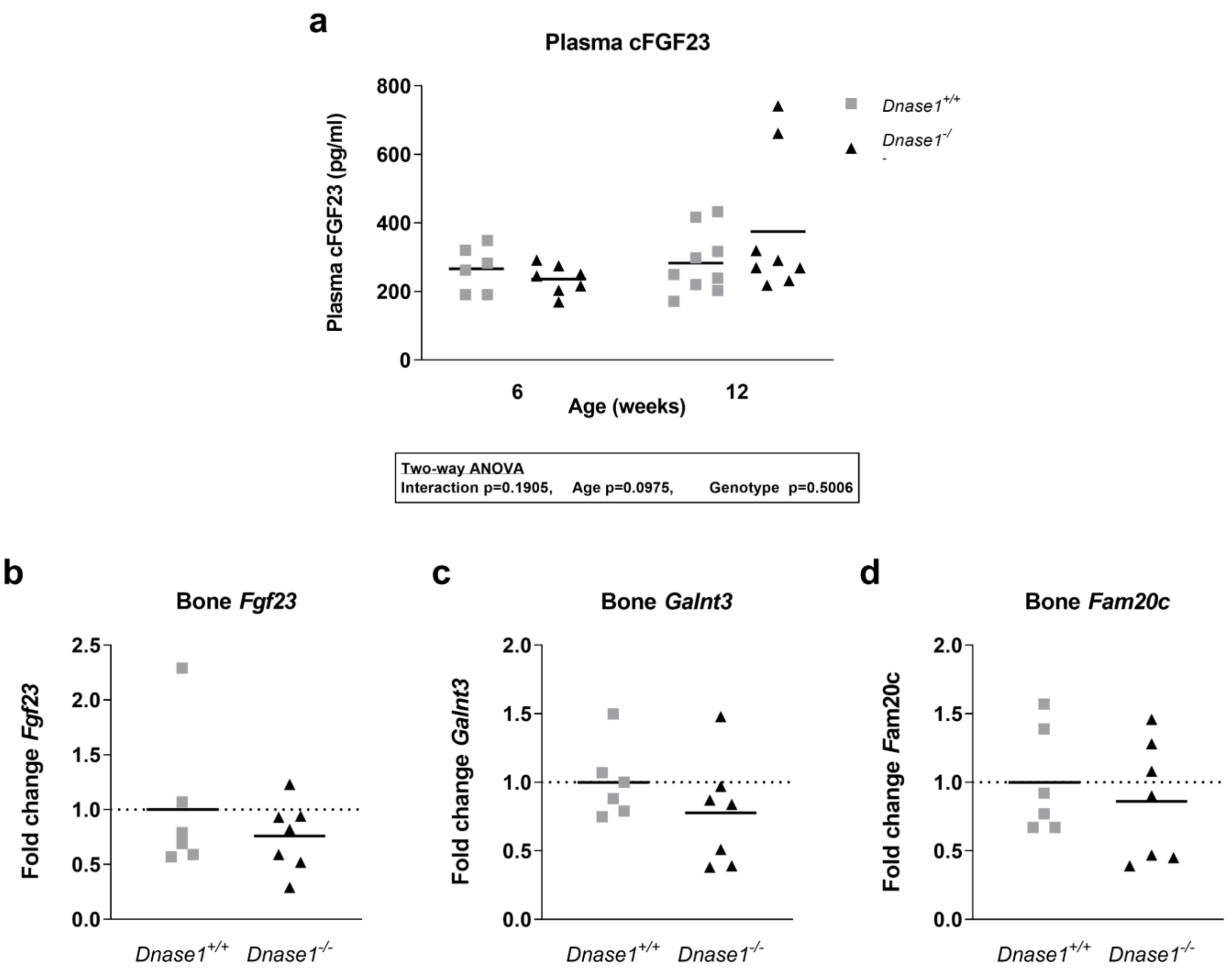

Supplementary Fig. S2. cFGF23 and *Fgf23* mRNA expression in bone of *Dnase1*$^{-/-}$ mice. Plasma (a) cFGF23 levels of 6 and 12 week old *Dnase1*$^{+/+}$ (■) and *Dnase1*$^{-/-}$ (▲) mice. 5-10 mice per group. Two-way ANOVA with Sidak's multiple comparisons test between genotypes, * $p < 0.05$. Relative (b) *Fgf23*, (c) *Galnt3*, and (d) *Fam20c* expression in bone in 6 week old *Dnase1*$^{+/+}$ (■) and *Dnase1*$^{-/-}$ (▲) mice. *18SrRNA* was used as housekeeping gene and values were normalized to vehicle group. 6-7 mice per group. Student's t-test, * $p < 0.05$.

Supplementary Figure S3

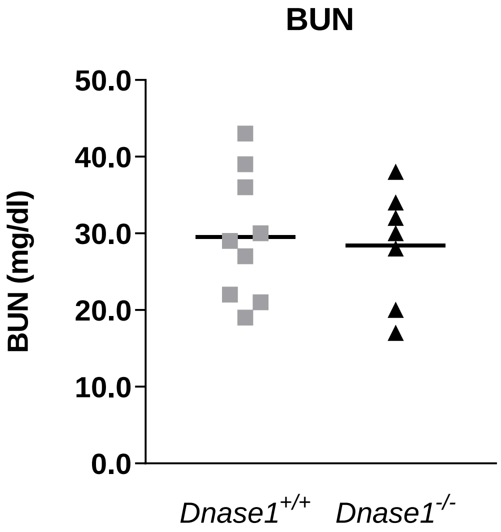

Supplementary Fig. S3. BUN in *Dnase1*$^{-/-}$ mice. Plasma BUN in 12 week old *Dnase1*$^{+/+}$ (■) and *Dnase1*$^{-/-}$ (▲) mice. 7-9 mice per group. Student's t-test, * $p < 0.05$.

Supplementary Figure S4

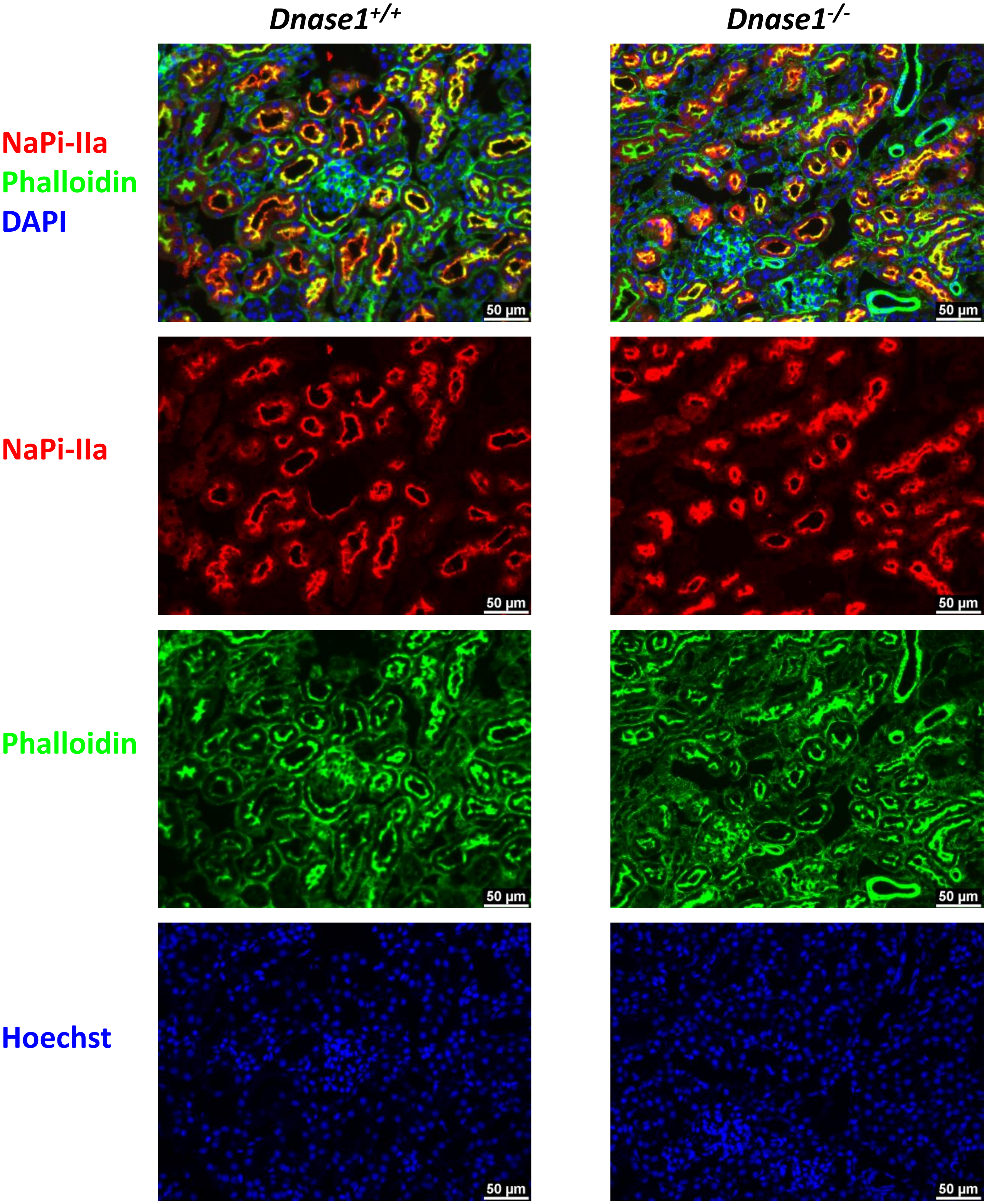

Supplementary Fig. S4. NaPi-IIa protein expression in *Dnase1*$^{-/-}$ mice. Immunohistochemistry for NaPi-IIa (red), phalloidin (green), and Hoechst (blue, cell nuclei). in 6 week old *Dnase1*$^{+/+}$ and *Dnase1*$^{-/-}$ mice. Original magnification 200x.

Supplementary Figure S5

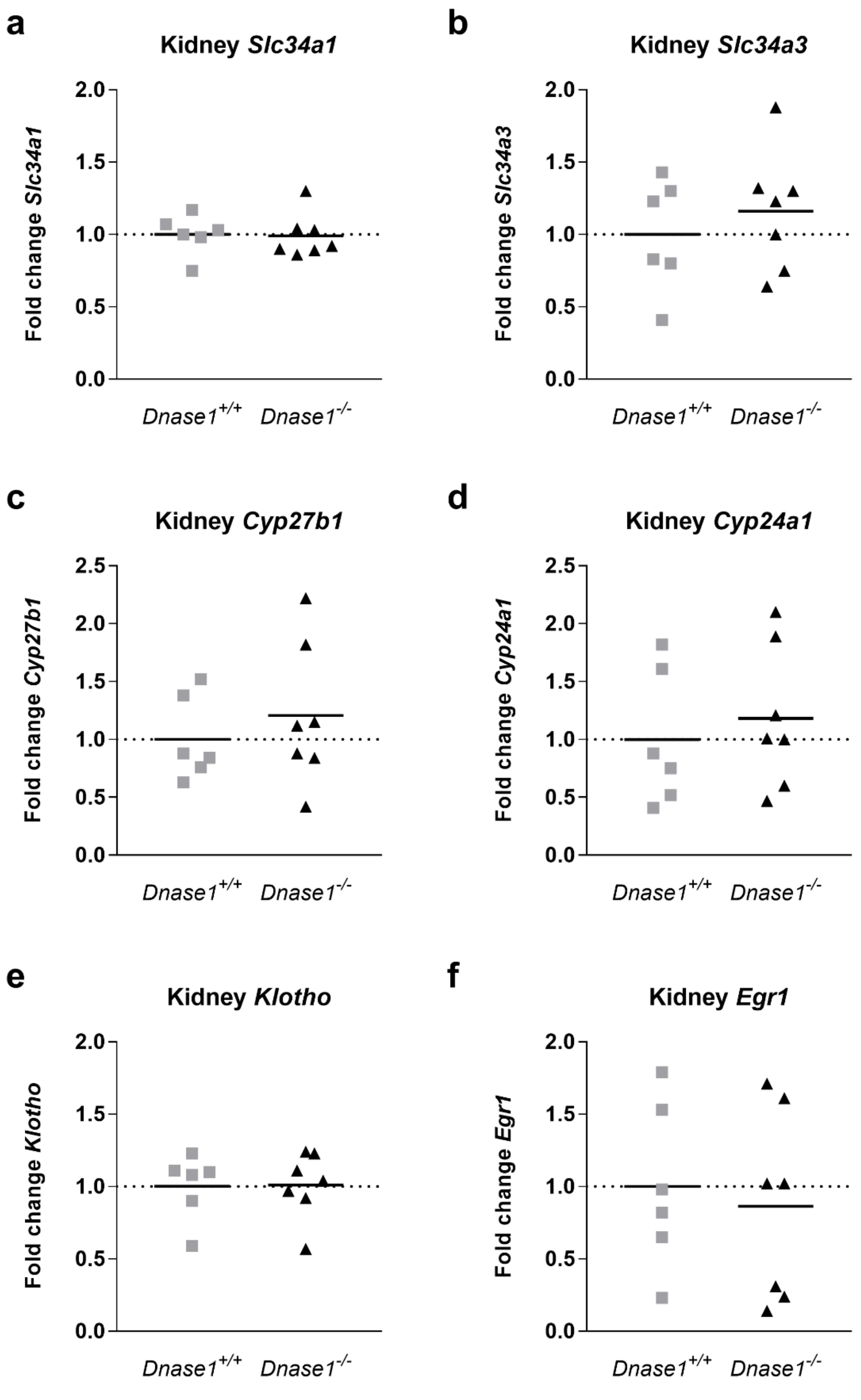

Supplementary Fig. S5. Renal mRNA expression of sodium phosphate co-transporter and 1,25(OH)$_2$D metabolizing enzymes in *Dnase1$^{-/-}$* mice. Relative renal (a) *Slc34a1*, (b) *Slc34a3*, (c) *Cyp27b1*, (d) *Cyp24a1*, (e) *Klotho,* and (f) *Egr1* mRNA expression in 6 week old *Dnase1$^{+/+}$* (■) and *Dnase1$^{-/-}$* (▲) mice. *18SrRNA* was used as housekeeping gene and values were normalized to vehicle group. 6-7 mice per group. Student's t-test, * $p < 0.05$.

Supplementary Figure S6

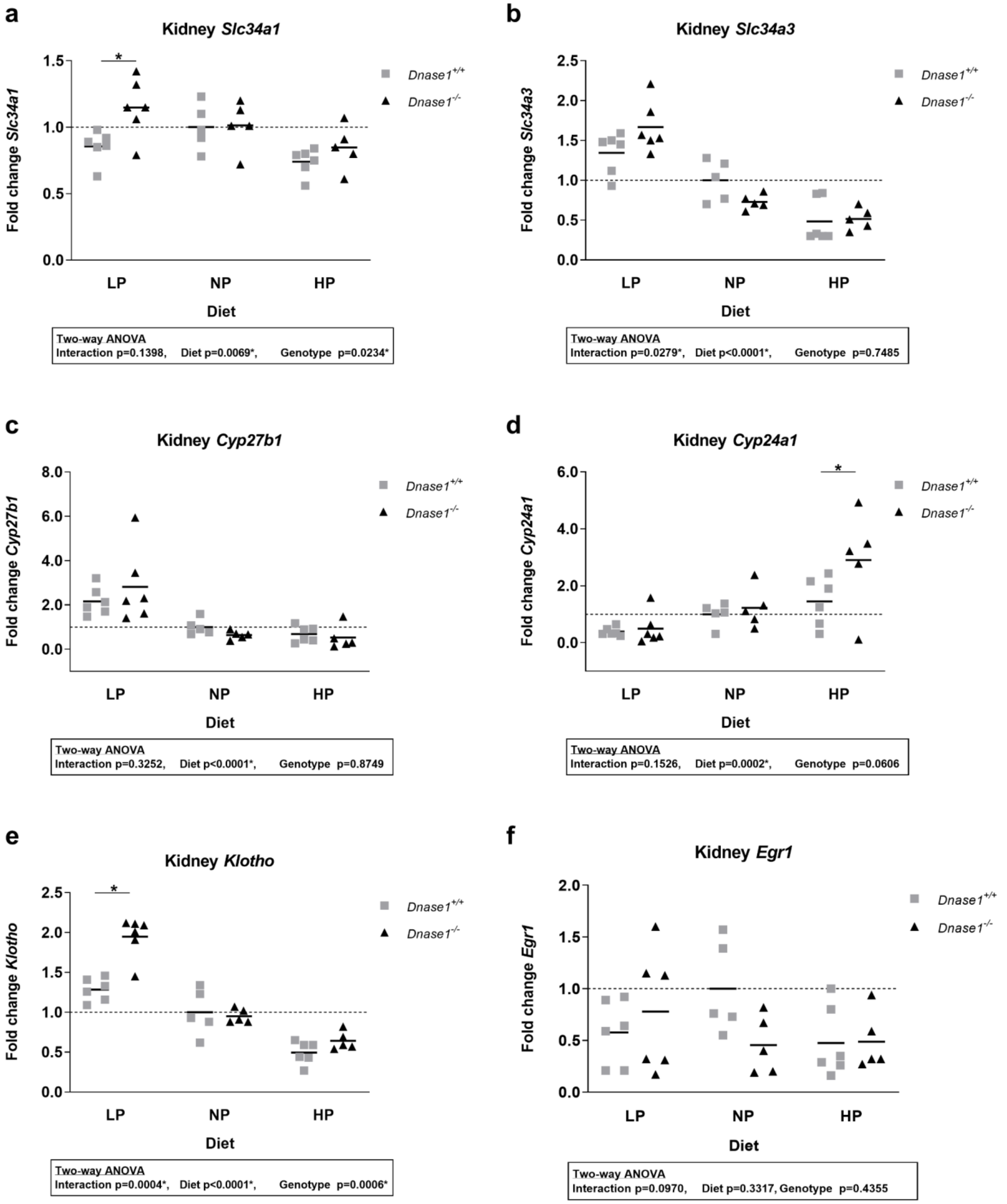

Supplementary Fig. S6. Renal mRNA expression of FGF23 regulated genes in *Dnase1*$^{-/-}$ mice. Relative renal (a) *Slc34a1*, (b) *Slc34a3*, (c) *Cyp27b1*, (d) *Cyp24a1*, (e) *Klotho and* (f) *Egr1* mRNA expression in 12 week old *Dnase1*$^{+/+}$ (■) and *Dnase1*$^{-/-}$ (▲) mice fed with low, normal or high phosphate diet for 4 days . *18SrRNA* was used as housekeeping gene and values were normalized to vehicle group. 5-6 mice per group. Two-way ANOVA with Sidak's multiple comparisons test between genotypes, * $p < 0.05$.

Supplementary Figure S7

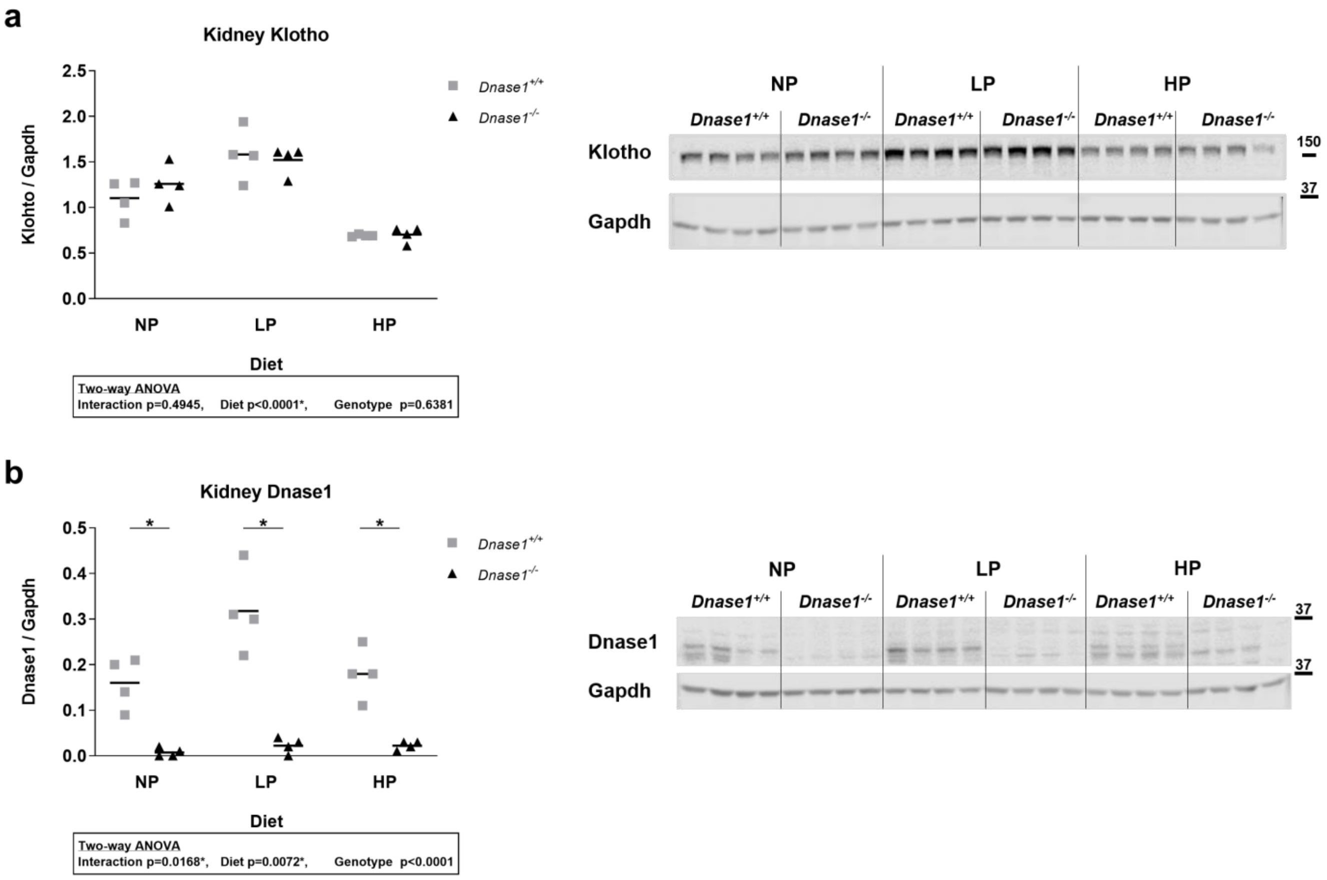

Supplementary Fig. S7. Klotho and Dnase1 protein expression in kidneys of *Dnase1$^{-/-}$* mice. (a) Renal Klotho and (b) Dnase1 protein expression relative to β-actin in 12 week old *Dnase1$^{+/+}$* (■) and *Dnase1$^{-/-}$* (▲) mice fed for 4 days with normal, low or high phosphate diet. 4 mice per group. Two-way ANOVA with Sidak's multiple comparisons test between genotypes, * $p < 0.05$. Full-length blots are presented in the Supplementary File 2.
