# Supplementary material for: Renal Dnase1 expression is regulated by FGF23 but loss of Dnase1 does not alter renal phosphate handling
Source: Sci Rep. 2021 Mar 17;11:6175. doi: 10.1038/s41598-021-84735-3 (PMC7969776; doi:10.1038/s41598-021-84735-3)

Renal *Dnase1* expression is regulated by FGF23 but loss of *Dnase1* does not alter phosphate metabolism in the kidney

Daniela Egli-Spichtig[1], Martin Y H Zhang[1], Alfred Li[2], Eva Maria Pastor Arroyo[3], Nati Hernando[3], Carsten A Wagner[3], Wenhan Chang[2], Farzana Perwad[1]

[1]Department of Pediatrics, Division of Nephrology, University of California San Francisco, USA

[2]San Francisco Veterans Affairs Medical Center (VAMC), Department of Medicine, University of California San Francisco, USA

[3]Institute of Physiology, University of Zurich, Zurich Switzerland and National Center of Competence in Research NCCR Kidney.CH, Switzerland

Corresponding author:

Farzana Perwad M.D.

University of California San Francisco

Children's Renal Center

550, 16th Street

5th Floor, MH-5351

San Francisco, CA-94143-3214

Ph: 415-476-2423

Fax: 415-476-7796

Email: farzana.perwad@ucsf.edu

Full size Western blot Figure 3a

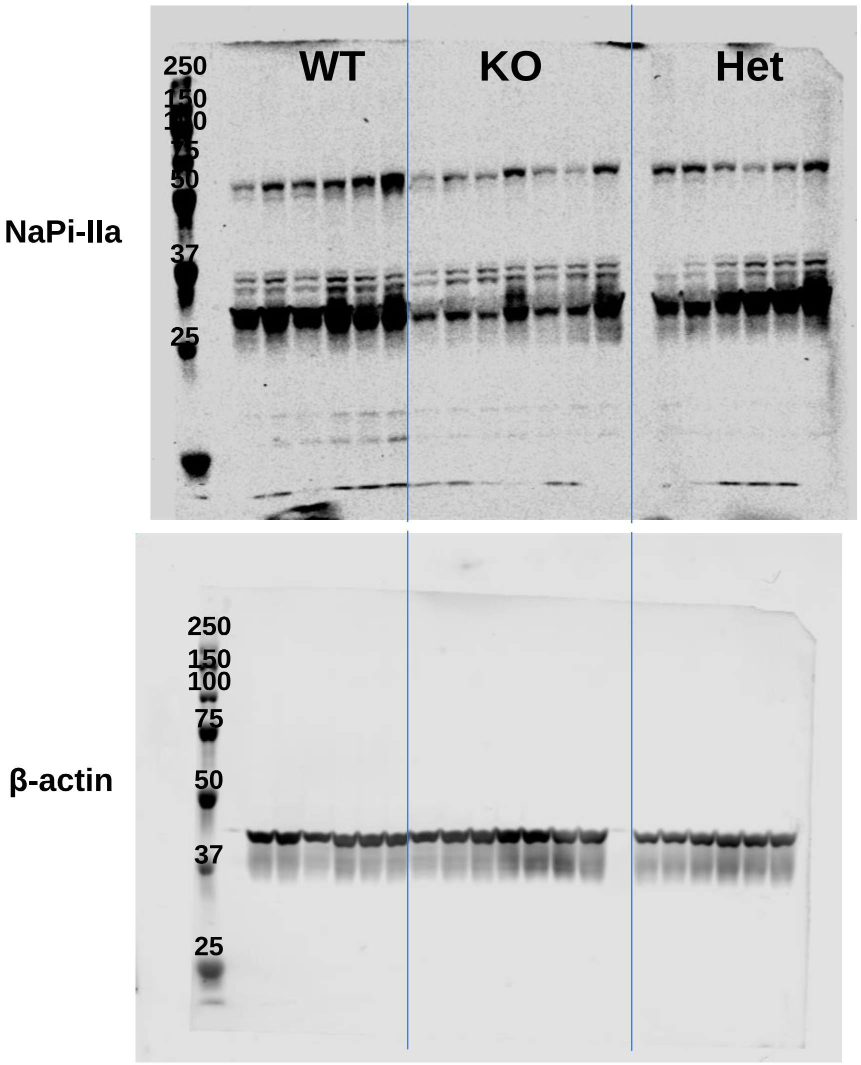

Full size Western blot Figure 3b

NaPi-IIc

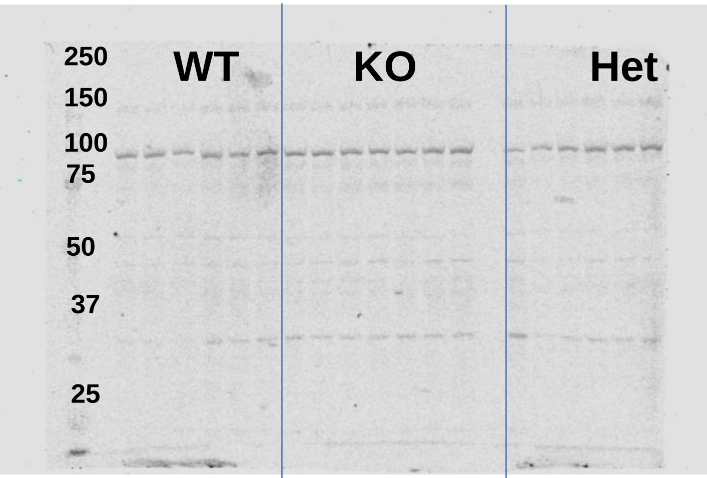

β-actin

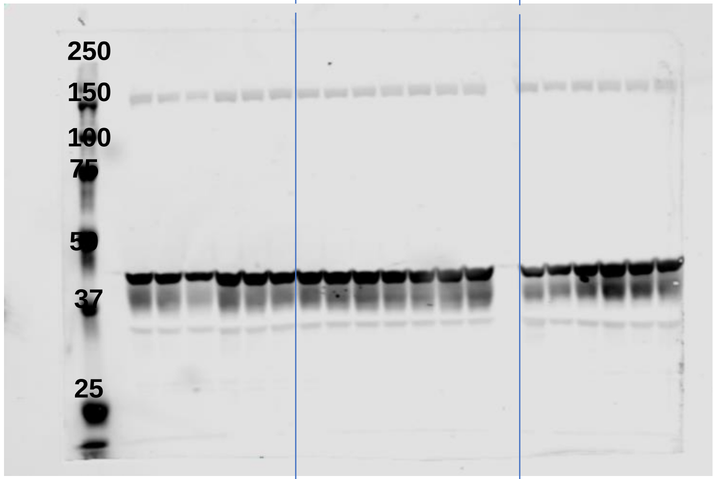

Full size Western blot Figure 3c

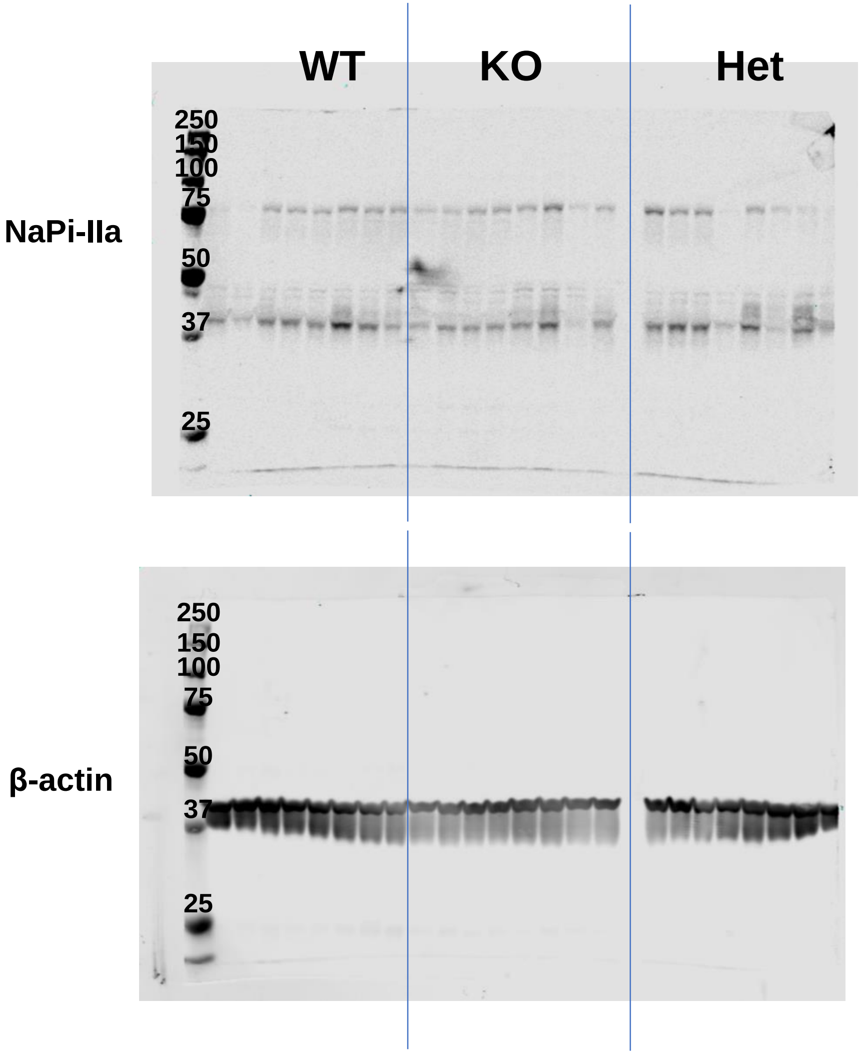

Full size Western blot Figure 3d

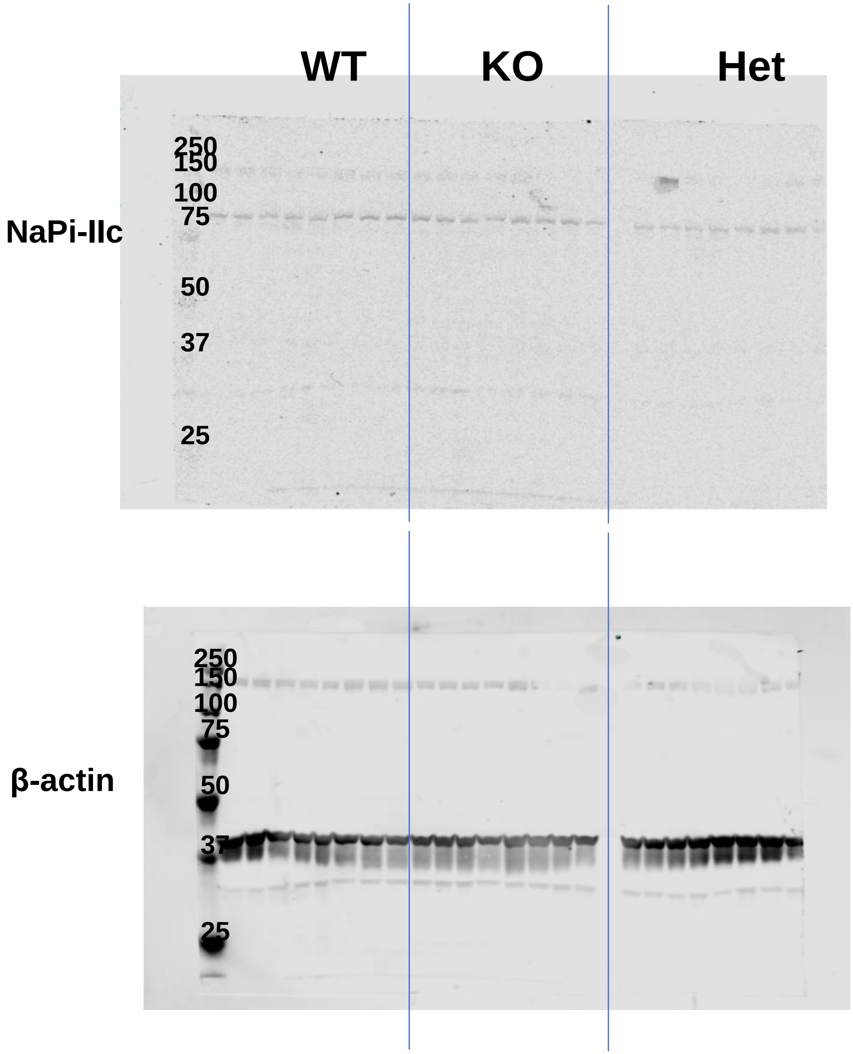

Full size Western blot Figure 4a

Klotho

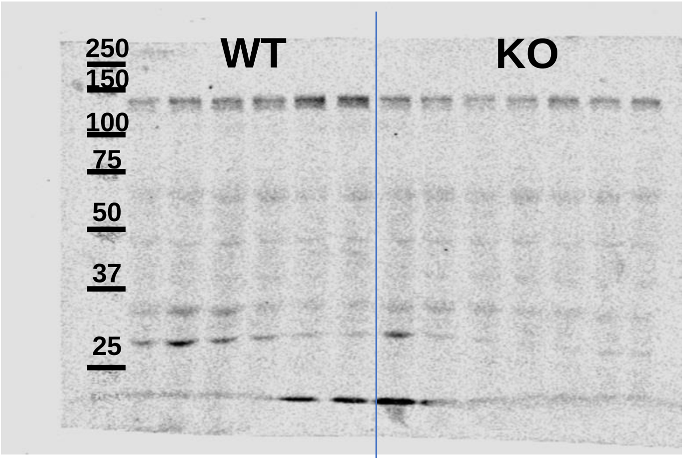

Gapdh

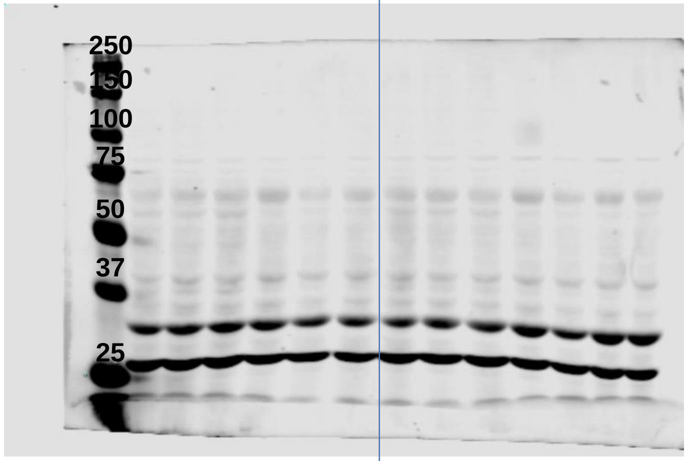

Full size Western blot Figure 4b

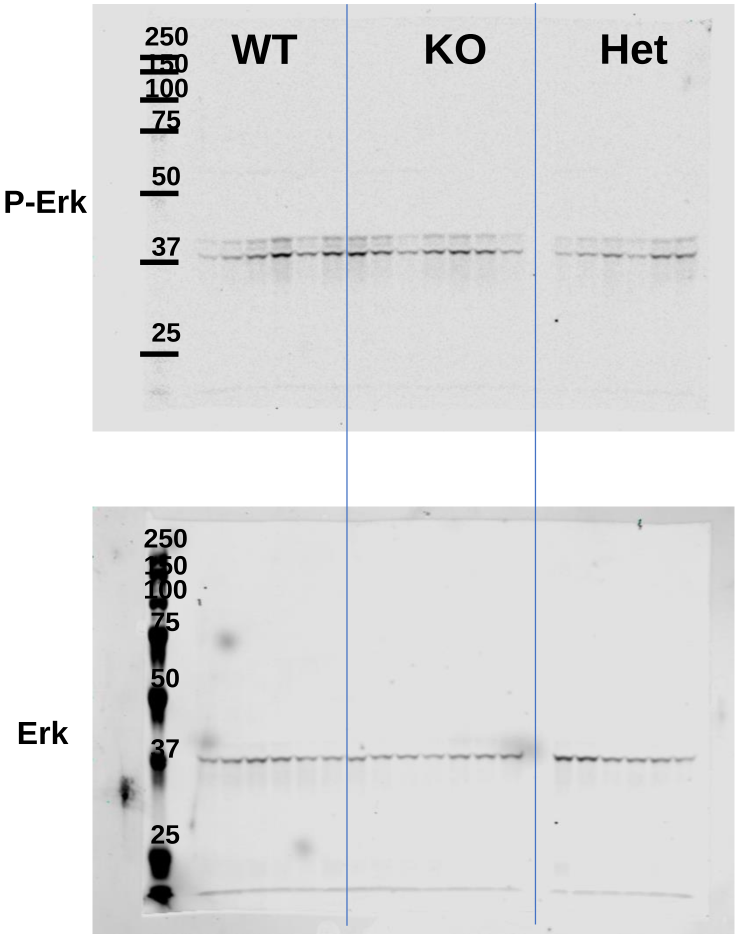

Full size Western blot Figure 6a

NaPi-IIa

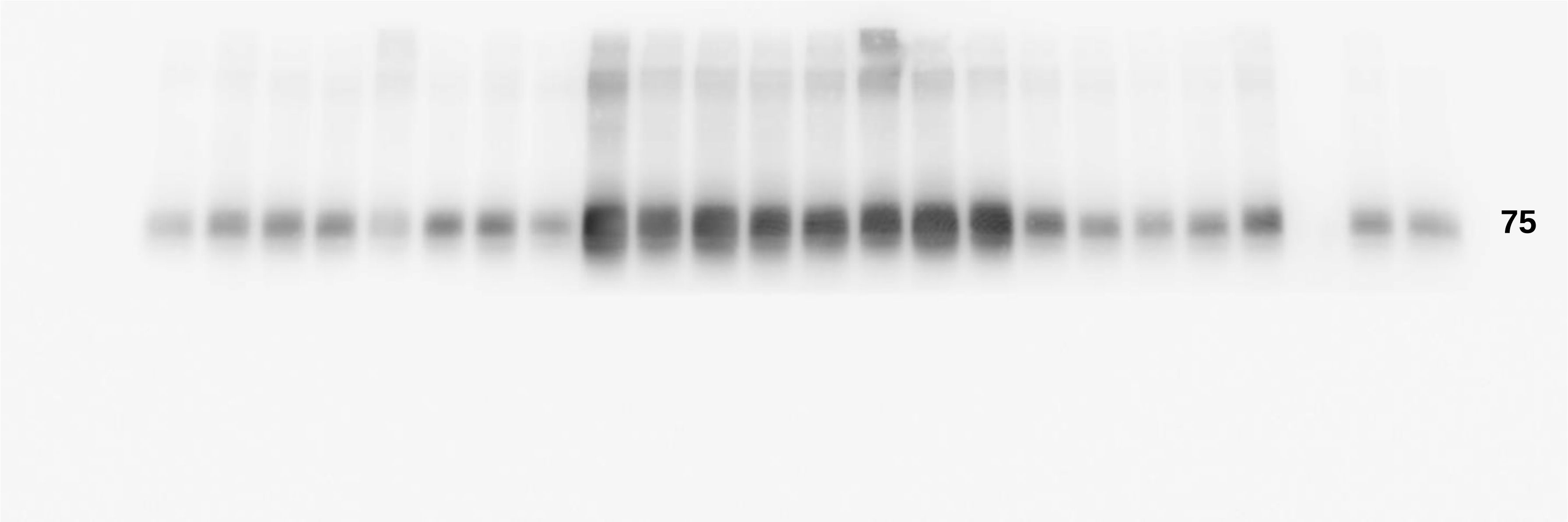

β-actin

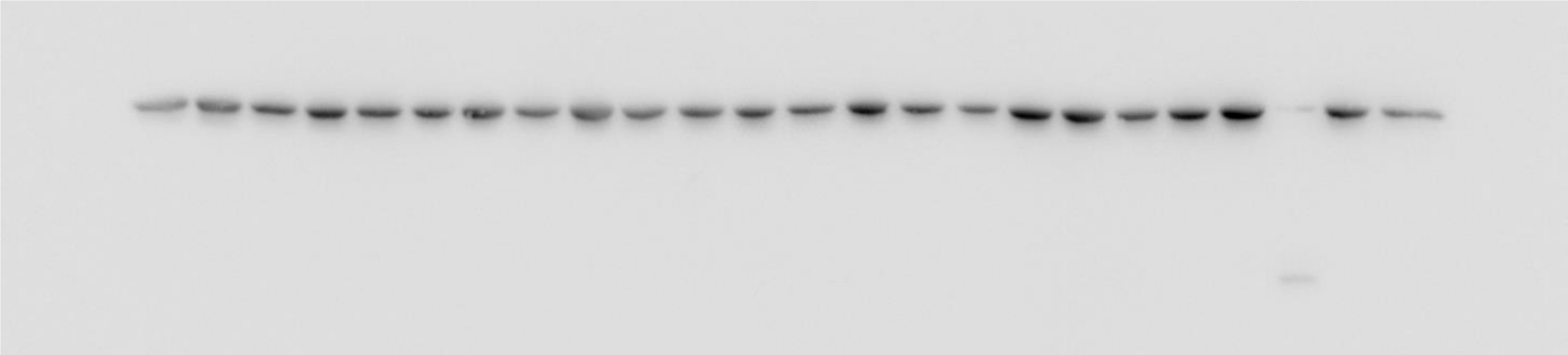

Full size Western blot Figure 6b

NaPi-IIc

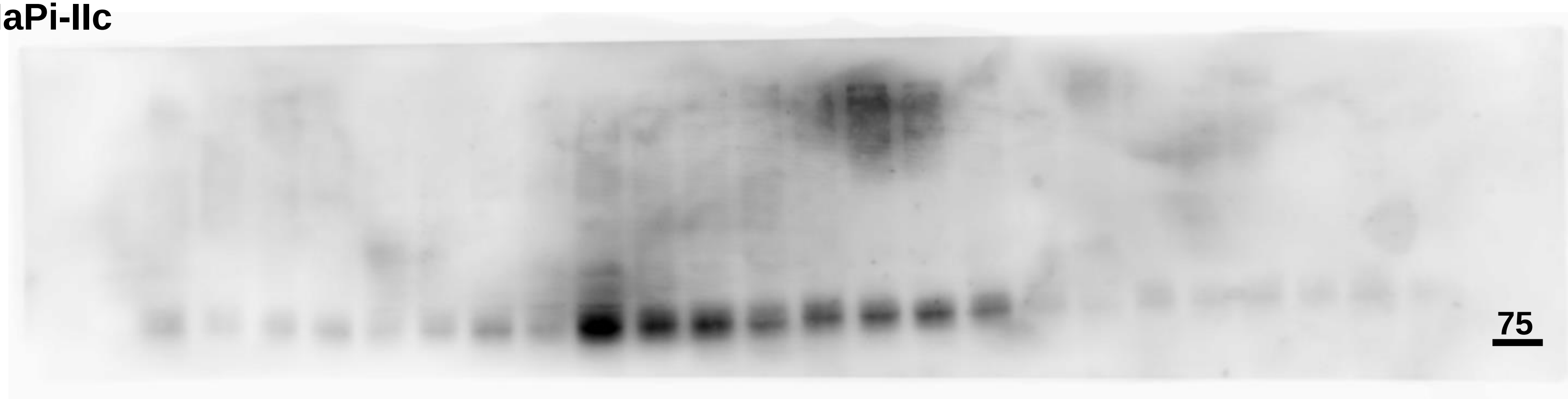

β-actin

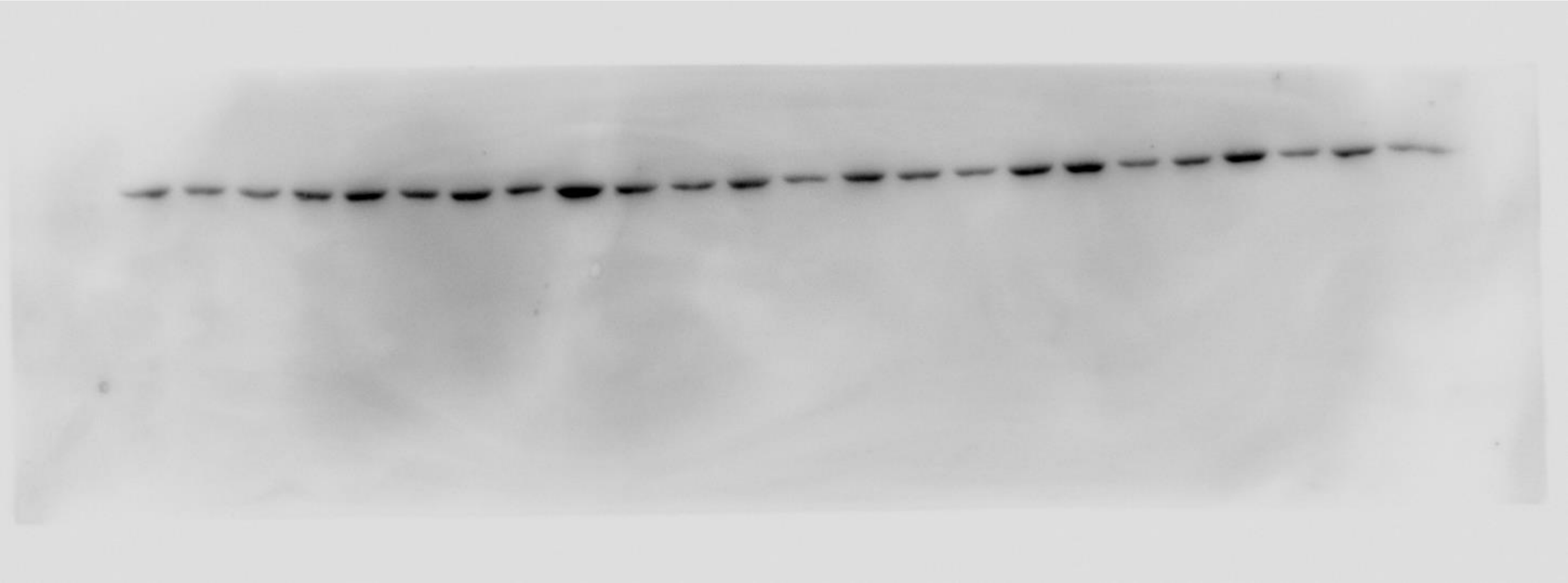

Full size Western blot Figure 7a low phosphate (LP)

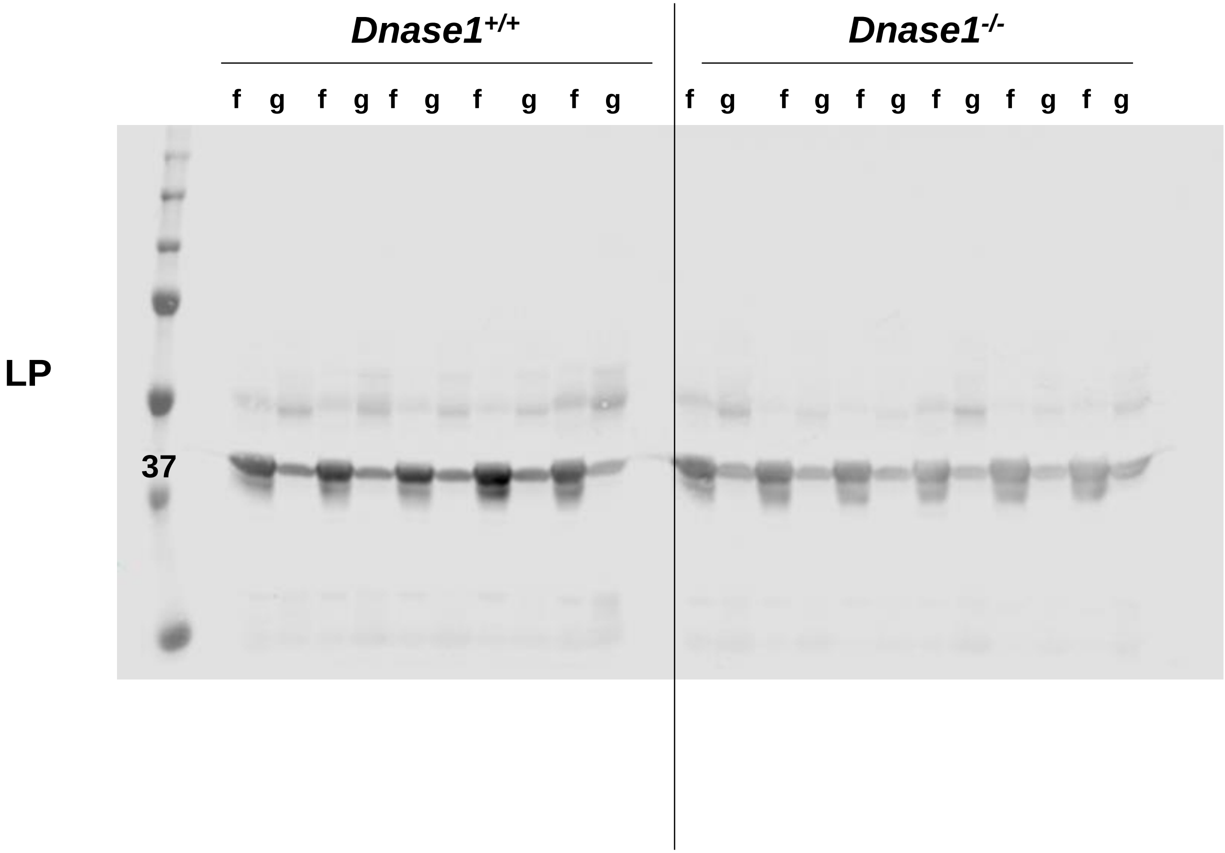

Full size Western blot Figure 7a normal phosphate (NP)

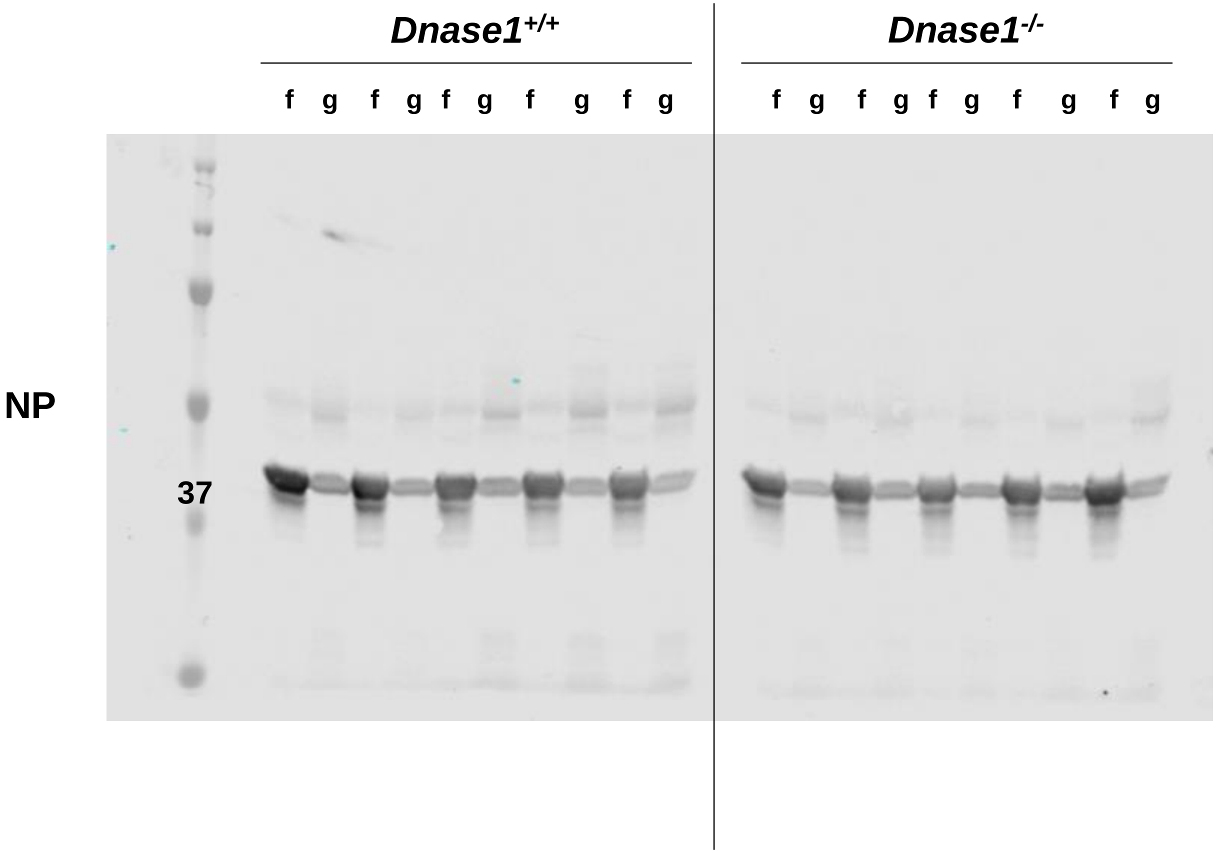

Full size Western blot Figure 7a high phosphate (HP)

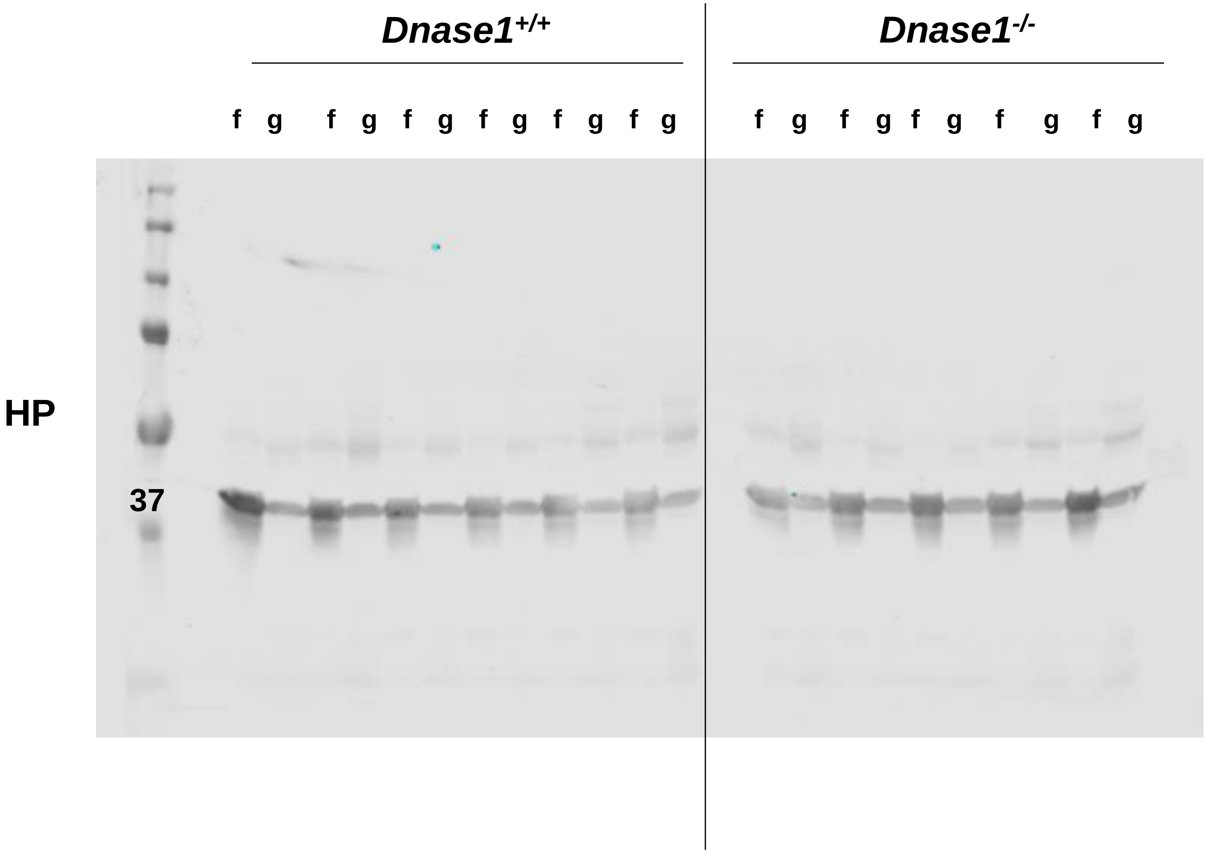

Full size Western blot Supplementary Figure 4a

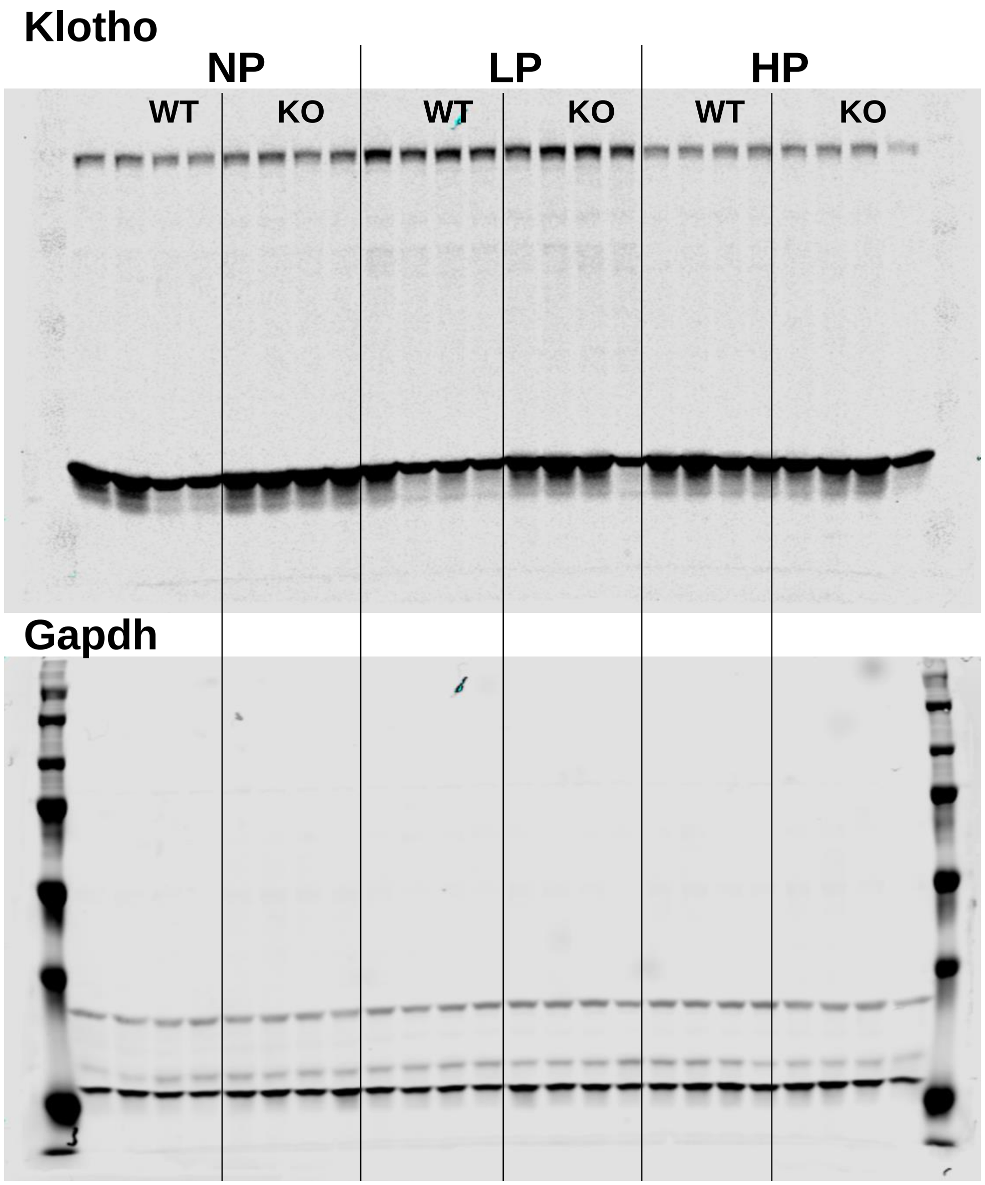

Full size Western blot Supplementary Figure 4b

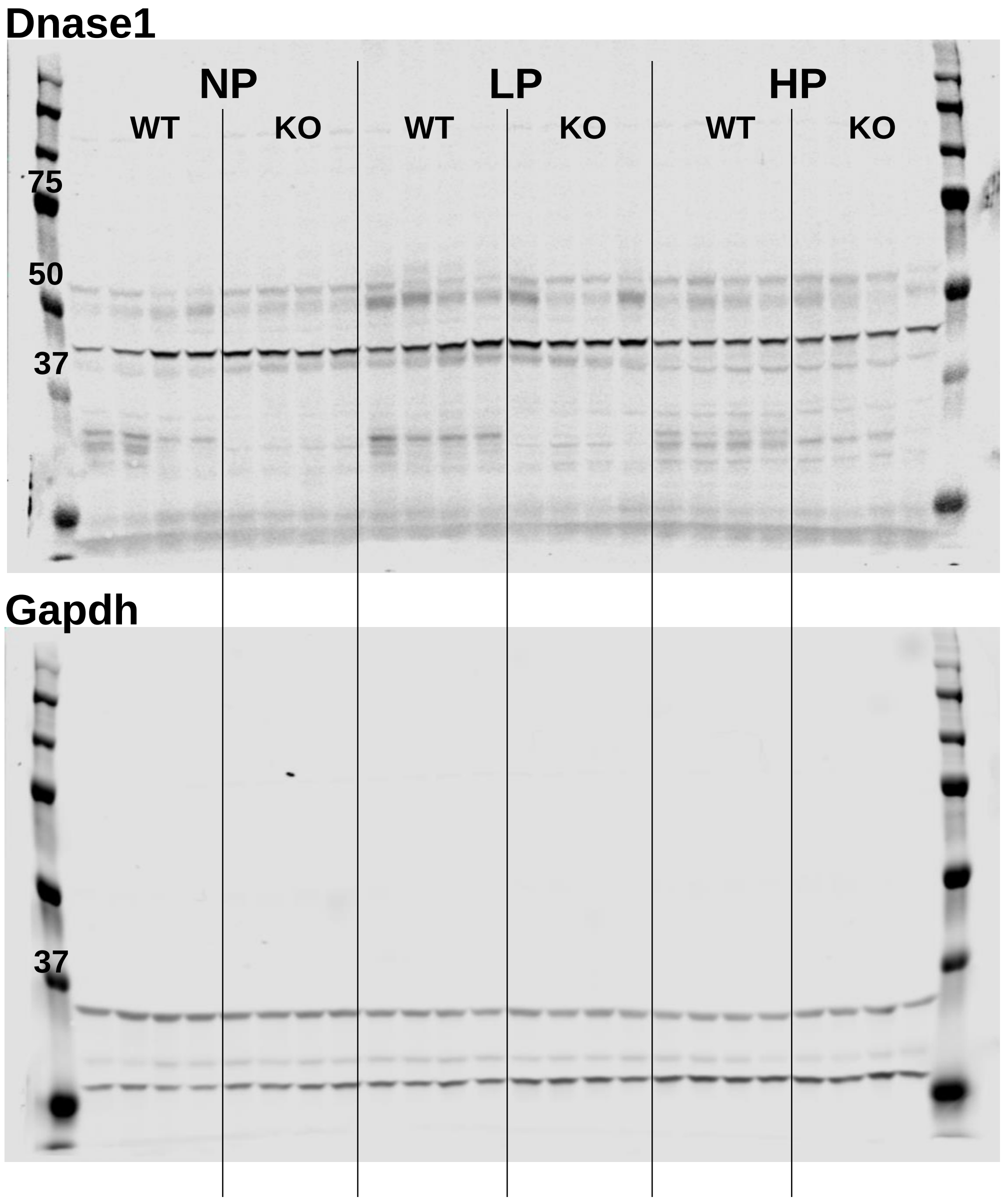

Supplement: Supplementary file 2 — Supplementary Information 2. [file 41598_2021_84735_MOESM2_ESM.pdf]
